# An assessment of the performance of the logistic mixed model for analyzing binary traits in maize and sorghum diversity panels

**DOI:** 10.1371/journal.pone.0207752

**Published:** 2018-11-21

**Authors:** Esperanza Shenstone, Julian Cooper, Brian Rice, Martin Bohn, Tiffany M. Jamann, Alexander E. Lipka

**Affiliations:** Department of Crop Sciences, University of Illinois at Urbana-Champaign, Urbana, Illinois, United States of America; Washington State Univeristy, UNITED STATES

## Abstract

The logistic mixed model (LMM) is well-suited for the genome-wide association study (GWAS) of binary agronomic traits because it can include fixed and random effects that account for spurious associations. The recent implementation of a computationally efficient model fitting and testing approach now makes it practical to use the LMM to search for markers associated with such binary traits on a genome-wide scale. Therefore, the purpose of this work was to assess the applicability of the LMM for GWAS in crop diversity panels. We dichotomized three publicly available quantitative traits in a maize diversity panel and two quantitative traits in a sorghum diversity panel, and them performed a GWAS using both the LMM and the unified mixed linear model (MLM) on these dichotomized traits. Our results suggest that the LMM is capable of identifying statistically significant marker-trait associations in the same genomic regions highlighted in previous studies, and this ability is consistent across both diversity panels. We also show how subpopulation structure in the maize diversity panel can underscore the LMM’s superior control for spurious associations compared to the unified MLM. These results suggest that the LMM is a viable model to use for the GWAS of binary traits in crop diversity panels and we therefore encourage its broader implementation in the agronomic research community.

## Introduction

The genome-wide association study (GWAS) is one of the most widely used quantitative genetics analyses in agronomy due to its potential to unlock the genomic sources of phenotypic variation [1]. Used as a discovery tool, the GWAS utilizes genome-wide marker sets in diversity panels to search the genome for polymorphisms that are associated with a phenotype of interest [2]. A genomic mechanism underlying the ability of a GWAS to successfully identify marker-trait associations is linkage disequilibrium (LD), defined as the non-random association of alleles at different loci [3]. A GWAS uses statistical models to search for indirect associations between single nucleotide polymorphisms (SNPs) and the phenotype of interest, relying on the property of LD to infer the location of the causal variant. The most commonly used statistical approach for a crop GWAS is to fit a model at each marker, where the trait of interest is the response variable, and the additive effects of the tested marker are an explanatory variable. [1].

The unified mixed linear model (MLM; [4]), which uses fixed and random effects to control for population structure and familial relatedness, is one of the most widely used statistical models in crop GWAS [1]. This model specifically controls for population structure through the incorporation of fixed effect covariates (e.g., principal components from a principal component analysis of a genome-wide marker set measured in a diversity panel). To account for relatedness, the unified MLM includes the individuals as a random effect, and then an additive genetic relatedness matrix (i.e., a kinship matrix) is used to estimate the variance-covariance between the individuals. Although the unified MLM has successfully elucidated genomic signals for a wide variety of traits [5–7], it cannot be used to analyze every possible class of agronomic traits because it assumes that the error terms are normally distributed, mutually independent, and are homoscedastic. For example, when the unified MLM is applied to binary traits (e.g., “1” = diseased vs. “0” = not diseased), violations of these assumptions are commonplace. Such violations could ultimately result in an empirical type I error rate that is substantially different than what was intended by the researcher [8]. As a result, a mixed model appropriate for binary traits, for example the logistic mixed model (LMM) [9], is necessary to identify biologically important signals associated with binary traits.

An extensive amount of research has been conducted for quantitative trait locus (QTL) analysis of binary traits in biparental crosses and related experimental populations. Many of these approaches assume that there is an unobserved continuous trait (called the liability) that underlies the binary trait; individuals with liability values that exceed an unknown threshold have a value of “1” (instead of “0”) for the binary trait [10]. The likelihood of the liability is then incorporated into approaches (described in e.g., [11–12]) similar to composite interval mapping [13–14] that seek to identify QTLs associated with binary traits. These approaches have been expanded upon to include Bayesian variants [15], to incorporate multi-family crosses [16–17], to analyze other types of discrete traits [18], and even to replace the likelihood of the liability with the probability that the binary trait equals “1” [19]. Collectively, this research sets a precedent into the amount of quantitative genetics and statistical theory, as well as utilization of the characteristics of the data set being analyzed, that should be investigated when applying statistical approaches for binary trait GWAS in crop diversity panels.

Important strides in the development of statistical approaches to analyze binary traits and to account for spurious associations have also been made outside the realm of QTL analyses. For example a considerable amount of work has been done in human case-control studies (e.g., [20]), which by design analyze binary traits. Statistical approaches designed to quantify the effects of genomic loci associated with disease status (a binary trait) have ranged in complexity from conducting a Pearson chi-square test at each SNP [21], to fitting a logistic regression model at each SNP [22], and even to using a mixed linear model to estimate the total amount of variation in the liability underlying the binary trait that is attributable to a tested genome-wide marker set [23]. The usefulness of mixed models to account for spurious associations has also been identified in association analyses across many species, and several studies have investigated the adaption of mixed models to analyze non-normally distributed traits [24–26]. Of all this work, the approach that is most directly applicable for analyzing binary traits in crop diversity panels is the generalized linear mixed model association test (GMMAT) [8]. Analogous to the genome-wide efficient mixed-model association approach for quantitative traits [27], GMMAT fits the computationally intensive LMM once, and then conducts a score test at each SNP to identify genomic loci associated with the binary trait under study. Given that the computationally efficient GMMAT is publicly available in the GENetic EStimation and Inference in Structured samples (GENESIS) [28] R package and that the LMM is theoretically optimal for the GWAS of binary agronomic traits, there is a critical need to use GMMAT to evaluate the performance of the LMM using actual data from crop diversity panels.

The purpose of this study was to compare the performance of the LMM to the unified MLM when analyzing binary traits in real crop diversity panels. To achieve this, we dichotomized three quantitative traits in a maize diversity panel and two quantitative traits in a sorghum diversity panel, and then conducted a GWAS on these dichotomized traits using both the LMM and the unified MLM. We also simulated an additional two binary traits using the maize data set to explore how well the LMM and unified MLM control the type I error rate in the presence of subpopulation structure. The objectives of this work were to i.) assess whether or not the LMM is capable of identifying the same peak marker-trait associations as those reported in the analysis of the original quantitative traits, and ii.) use agronomic data to determine if the LMM is capable of superior control of spurious associations for binary traits where the observed proportion of “1’s” differ across subpopulations, as was originally demonstrated using human case-control and simulated data in [8]. We were consequently able to document the applicability and usefulness of the LMM for analyzing binary agronomical traits.

## Materials and methods

### Description of phenotypic and genotypic data

We used publicly available phenotypic and genotypic data from two crop diversity panels to conduct our analyses. One of these diversity panels consists of maize lines, while the other consists of sorghum lines. To assess the performance of the LMM for binary traits where there were roughly an equal number of observed “0’s” and “1’s”, as well as for binary traits where there was an unequal number of “0’s” and “1’s”, we dichotomized (i.e., converted to binary traits) each of five studied quantitative traits twice. First, for a given quantitative trait, all lines with trait values greater than the 50^th^ percentile were given a value of “1”, while the remaining lines were given a value of “0”. The second dichotomization was conducted in a similar manner, except that all lines with trait values greater than the 75^th^ percentile were given a value of “1” and the remaining lines were given a value of “0”. Given that the observed proportion of “0’s” and “1’s” in a binary trait could theoretically range from [0,1], these two dichotomizations were essential for determining whether or not any advantages in the performance of the LMM for binary trait were specific to a particular observed ratio of “0’s” and “1’s”. All genotypic, phenotypic, and R scripts used in this work are available at: https://figshare.com/articles/Shenstone_et_al_2018_zip/7212902.

### Goodman maize diversity panel

In its entirety, the Goodman diversity panel [29] contains 302 unique maize lines and captures 75% of all allelic diversity in maize [30]. As such, this panel consists of lines from various subpopulations of maize, including stiff stalk, non-stiff stalk, tropical, and popcorn lines. We dichotomized three publicly available quantitative traits measured in various subsets of lines in this panel. The first, *α*-tocopherol grain content, was measured in the subset of 252 lines originally published in [31]. Hypothesized to have a relatively tractable genetic architecture [31–32], previous studies of this trait in maize have identified peak marker-trait associations in the vicinity of the *ZmVTE4* tocochromanol biosynthetic pathway gene on chromosome 5 [31–33]. The second trait from this panel dichotomized for this study was zeaxanthin levels measured in the grain of a subset of 201 lines with non-white kernels, which was originally published in [34]. Although this trait is hypothesized to be controlled by a small number of genes, the strength of the peak marker-trait associations identified for this trait (analyzed in [34]) were not as strong as those identified for *α*-tocopherol. The final trait we dichotomized in this panel was ear height (http://www.maizegenetics.net/tassel) measured in a subset of 278 lines. A previous GWAS of this trait in the Goodman diversity panel [35] did not identify any statistically significant marker-trait associations.

The genotypic data used in this study have been extensively documented elsewhere (e.g., [34] and [35]). In total, between 294,290–299,723 SNPs obtained from the Illumina MaizeSNP50 BeadChip ([36], available at https://cbsusrv04.tc.cornell.edu/users/panzea/download.aspx?filegroupid=7), genotyping-by-sequencing ([37], available at https://cbsusrv04.tc.cornell.edu/users/panzea/download.aspx?filegroupid=5) and various other genotyping technologies [4,37] were used in the analysis of these data. As done in previous studies (e.g., [31,34]), the missing values of all SNPs were imputed with the major allele.

### US sorghum association panel

We analyzed a total of 320 accessions from the US sorghum association panel [38]. Similar to the Goodman maize diversity panel, this sorghum diversity panel contains accessions that are representative of the entirety of sorghum diversity. Both of the quantitative traits that we dichotomized were analyzed and published in [38] and [39]. The first of these traits, plant height, is considered to be polygenic. The GWAS conducted by [38] underscores this conjecture by identifying two genomic regions exhibiting peak marker-trait associations. In contrast the second trait, branch length, is thought to be more complex, and the corresponding GWAS did not identify as strong marker-trait associations. We used a total of 115,767 GBS SNPs originally from [40] in our analyses. Consistent with the analysis of the Goodman maize diversity panel, all missing genotypic data were imputed with the major allele.

### Statistical models considered for GWAS

#### Logistic mixed model (LMM)

The GMMAT procedure used to fit the LMM at each dichotomized trait and then use the score test to test for a statistically significant marker-trait association, as has been previously described [8]. Within the context of the work presented in this paper, the LMM used to associate each marker with each dichotomized trait is presented as follows:

*Y*_*i*_ *are independent Bernoulli random variables with expected values*:

$$E\left\{Y_{i}\right\}=\pi_{i}$$

*and variance of*:

$$Var\left\{Y_{i}\right\}=\pi_{i}\left(1-\pi_{i}\right)$$

*and*:

$$\text{log}\;\left(\frac{\pi_{i}}{1-\pi_{i}}\right)=\mu +\Sigma_{k=1}^{3}\beta_{k}PC_{ik}+\alpha x_{i}+Line_{i},$$

*and*:

*π*_*i*_ = *P*{*Y*_*i*_ = 1} = *probabiltiy that the binary trait at the i*^*th*^ *line takes on a value of* 1,

*μ* = *the grand mean*,

*β*_*k*_ = *fixed effect of the k*^*th*^ *principal component* (*PC*),

*PC*_*ik*_ = *value of the k*^*th*^ *PC for i*^*th*^ *line*,

*α* = *fixed additive effect of the tested marker*,

$\begin{array}{l} x_{i}=observed\;genotype\;of\;tested\;marker\;for\;the\;i^{th}\;line,\\ =\{\begin{array}{c} 0,\;if\;aa\\ 1,\;if\;Aa\;or\;aA\\ 2,\;if\;AA \end{array} \end{array}$,

*Line*_*i*_ = *Random effect of the i*^*th*^ *line*,

*and*:

$\left(Line_{1},\ldots ,Line_{n}\right)∼MVN\left(0,2K\sigma_{G}^{2}\right)$, *and*:

*K* = *kinship* (*i*.*e*. *additive genetic relatedness*) *matrix*

For the traits analyzed in maize, the PCs and kinship matrix described in [31] were used. Similarly, the 115,767 GBS SNPs from [40] were used to obtain the PCs and kinship matrix (calculated with the method described in [41]) used in the analysis of the sorghum data. For both panels, the first three PCs were determined to adequately control for population structure (S1 and S2 Figs), which is consistent with previous GWAS of both data sets (e.g.,[31,38]). The use of the score test by GMMAT substantially reduces computational burden because the LMM only needs to be fitted once (without any marker included as an explanatory variable) for a given GWAS [9]. The computational time required to run an LMM-based GWAS on these data using GMMAT on a MacBook Pro was similar to that required for running unified MLM-based GWAS (S1 Table). We used the Benjamini-Hochberg procedure [42] to control the false discovery rate (FDR) at 5% and 10%.

#### Unified MLM

For each dichotomized trait, a second GWAS was conducted using the unified MLM [4] with population parameters previously determined [43] in the Genome Association and Prediction Integrated Tool (GAPIT) R package [44]. Within each species, the same respective PCs and kinship matrix as those considered for the LMM were also used for this GWAS. Again, the FDR was controlled at 5% and 10% using the Benjamini-Hochberg procedure [42].

### Comparison of false positive control between the LMM and unified MLM

Using an asthma case-control study and simulated data, [8] demonstrated that when the prevalence of a binary trait (i.e., the probability that a binary trait equals “1”) substantially differs for at least one subpopulation of individuals in a data set, a GWAS using the unified MLM will inadequately control for type I errors. Moreover, this previous study showed that it is potentially difficult to identify such erroneous type I error rates using standard diagnostic approaches (e.g., a QQ-plot of −log(10) *P*-values or calculating a genomic control, or GC, statistic [45]) because SNPs that are more common in the subpopulation with higher prevalence tend to have more significant *P*-values than expected under *H*_0_: No marker-trait association from the unified MLM, while SNPs that were more rare in the same subpopulation tended to have less significant *P*-values under the same null hypothesis. Finally, [8] demonstrated that the LMM has superior control of the type I error rate regardless of the distribution of SNP alleles within the subpopulations. Given the importance of this result, we conducted an analysis similar to [8] on our data to determine if it is possible to observe inadequate control of the false positive rate when using the unified MLM to analyze binary traits in a crop diversity panel.

Due to the availability of information on its subpopulation structure, we chose to use the Goodman maize diversity panel for this analysis. Based on our observation that tropical lines in this panel tended to have larger ear height than non-tropical lines, we decided that ear height dichotomized at the 75^th^ percentile would be the ideal trait to analyze. That is, we hypothesized that we would observe a substantially higher proportion of “1’s” for this trait in the tropical subpopulation compared to other subpopulations. Thus for this trait, we filtered out all 32,110 SNPs that were monomorphic within either of these two subpopulations (i.e., tropical and non-tropical) and assessed the distributions of test statistics and *P*-values from the remaining 262,191 SNPs. Following the analysis of [8], we used minor allele frequencies (MAFs) to subdivide these SNPs by the expected variance of allele frequencies [2MAF(1-MAF)] within these two subpopulations as follows: i.) all SNPs where the ratio of expected variance between tropical and non-tropical was less than 0.80 (i.e., SNPs that tended to be more common in the non-tropical subpopulation), ii.) all SNPs where the ratio was between 0.80 and 1.25 (SNPs that have similar allele frequencies in both subpopulations), and iii.) all SNPs where this ratio was greater than 1.25 (SNPs that tended to be more common in the tropical subpopulations). We then used QQ-plots of the −log(10) *P*-values and GC of SNP test statistics within and across these three subdivisions of SNPs to assess the extent to which the unified MLM and LMM were controlling for spurious associations. In general, GC inflation factors [45], estimated as a function of the median of the test statistics across all SNPs, that are close to *λ* = 1 suggests that test statistic values are not unduly inflated (or deflated) by population structure.

To further explore the influence of unequal proportion of “1’s” of a binary trait among these two subpopulations on the empirical null distribution of *P*-values from the LMM and the unified MLM, we repeated the above analysis on two binary traits simulated on the same all *n* = 278 maize lines with ear height data. The first binary trait was generated by simulating Bernoulli(*π* = 0.5) random variables for each of the 64 tropical maize lines and Bernoulli(*π* = 0.05) random variables on the 216 non-tropical maize lines, while the second binary trait was generated by simulating Bernoulli(*π* = 0.5) random variables on all 278 lines. Because there were no genetic components underlying these binary traits (i.e., no quantitative trait nucleotides were simulated), the resulting empirical distributions of *P*-values from the LMM and unified MLM were generated under *H*_0_: No marker-trait association. Thus, any deviations of these empirical *P*-values from the expected Uniform[0,1] distribution would suggest inadequate control of type I errors.

## Results

### Results for dichotomizing maize and sorghum quantitative traits at the 50^th^ percentile

**Fig 1:**
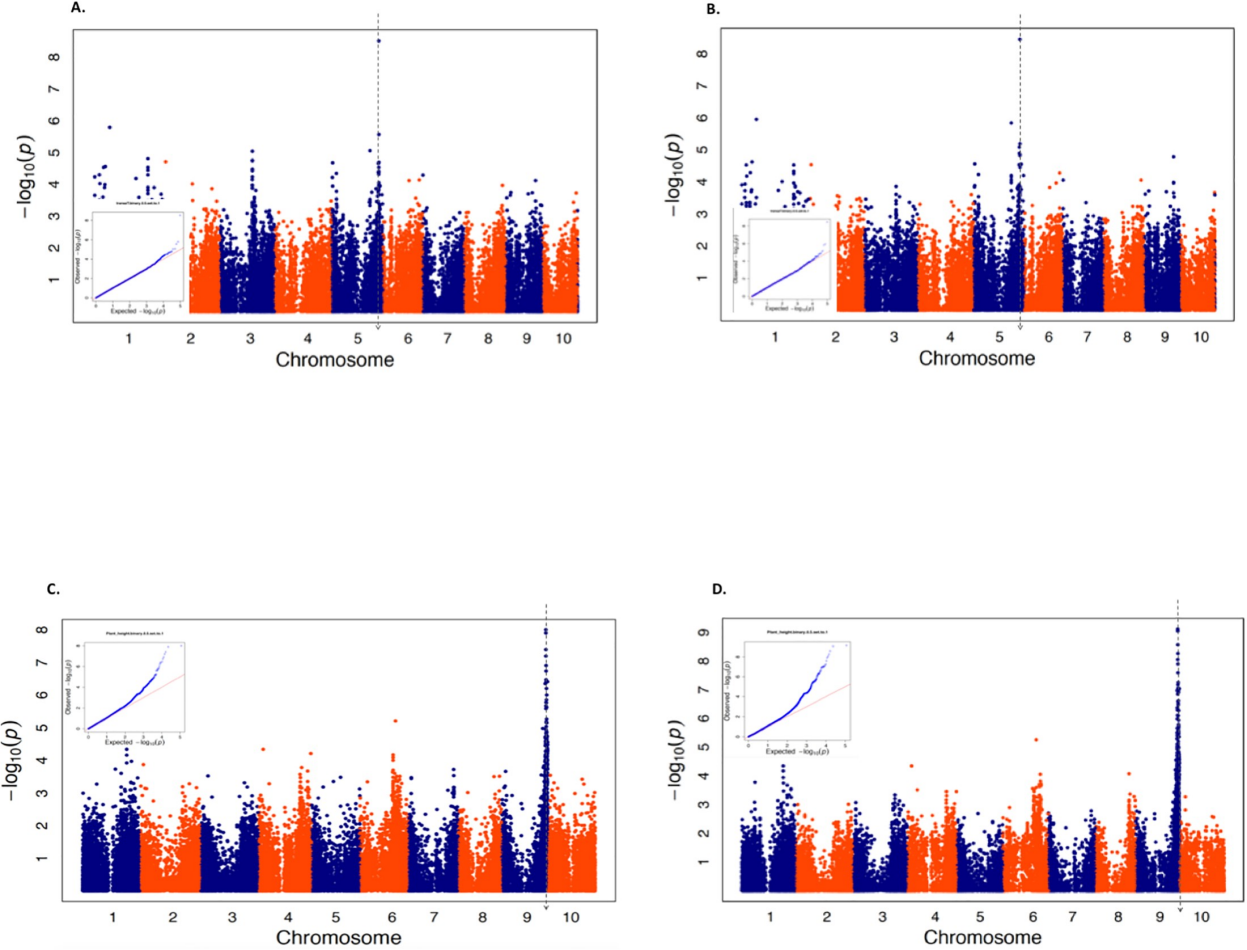
Results for two quantitative traits dichotomized at the 50^th^ percentile. Manhattan plots summarizing the genome-wide association study (GWAS) results for two quantitative traits dichotomized at the 50^th^ percentile. Each trait was analyzed using both the unified mixed linear model (MLM) and the logistic mixed model (LMM). Quantile quantile (QQ)-plots depicting the observed (Y-axis) and expected (X-axis) −log(10) *P*-values are inserted into each Manhattan plot. The Manhattan plots on each graph shows the physical bp position of each tested SNP in either the maize B73_RefGen v2 reference genome (for A and B) or the sorghum Btx623 v2.1 reference genome (for C and D) on the X-axis; while the −log(10) *P*-values from either the unified MLM (A and C) or LMM (B and D) on the Y-axis. The vertical line on each graph indicates the approximate location of the peak marker-trait associations identified in the previously-published GWAS of the original quantitative trait. (A) Results for a GWAS on dichotomized α-tocopherol measured in maize grain using the unified MLM. (B) Results for a GWAS on dichotomized α-tocopherol measured in maize grain using the LMM. (C) Results for a GWAS on dichotomized sorghum plant height using the unified MLM. (D) Results for a GWAS on dichotomized sorghum plant height using the LMM.

To assess the ability of the LMM to analyze binary agronomical traits, we dichotomized three quantitative traits in maize and two quantitative traits in sorghum based on the 50^th^ percentile of each trait. For each of these five dichotomized traits, we then used marker data in the respective maize and sorghum diversity panels to conduct a GWAS using two statistical models, specifically the LMM and the unified MLM. For each trait, the performance of each model was evaluated by assessing how well they controlled for spurious associations and how many statistically significant marker-trait associations that they identified. Both the LMM and the unified MLM appeared to adequately control for spurious associations when commonly used diagnostic approaches were implemented (Table 1, Fig 1 and S3–S8 Figs). We then compared the number of statistically significant associations identified by these two models at 5% and 10% FDR (Table 1). Interestingly for the two dichotomized traits where statistically significant associations were identified (*α*-tocopherol in maize and plant height in sorghum), either an equal or greater number of statistically significant associations were identified by the LMM. These significantly associated SNPs identified using both models colocalized to the same genomic regions harboring the most significant marker-trait associations in the published GWAS results of the original quantitative traits reported in [31], [33], [38] and [39](Fig 1).

**Table 1:** Genome-wide association study results for quantitative traits that are dichotomized at the 50^th^ percentile. Number of statistically significant marker-trait associations and genomic control values from the unified mixed linear model and the logistic mixed model are presented.

Species	Dichotomized Trait	No. of Significant Associations				GC^a^ *λ* Values	
		5% FDR^b^		10% FDR			
		MLM^c^	LMM^d^	MLM	LMM	MLM	LMM
Maize	*α*-tocopherol	3	3	3	5	1.03	1.03
Maize	Zeaxanthin	0	0	0	0	1.02	1.04
Maize	Ear Height	0	0	0	0	1.04	1.03
Sorghum	Plant Height	35	169	72	259	1.01	1.01
Sorghum	Branch Length	0	0	0	0	1.02	0.96

^***a***^GC, Genomic control

^*b*^FDR, False discovery rate

^*c*^MLM, Unified mixed linear model

^*d*^LMM, Logistic mixed model

### Results for dichotomizing maize and sorghum quantitative traits at the 75^th^ percentile

**Fig 2:**
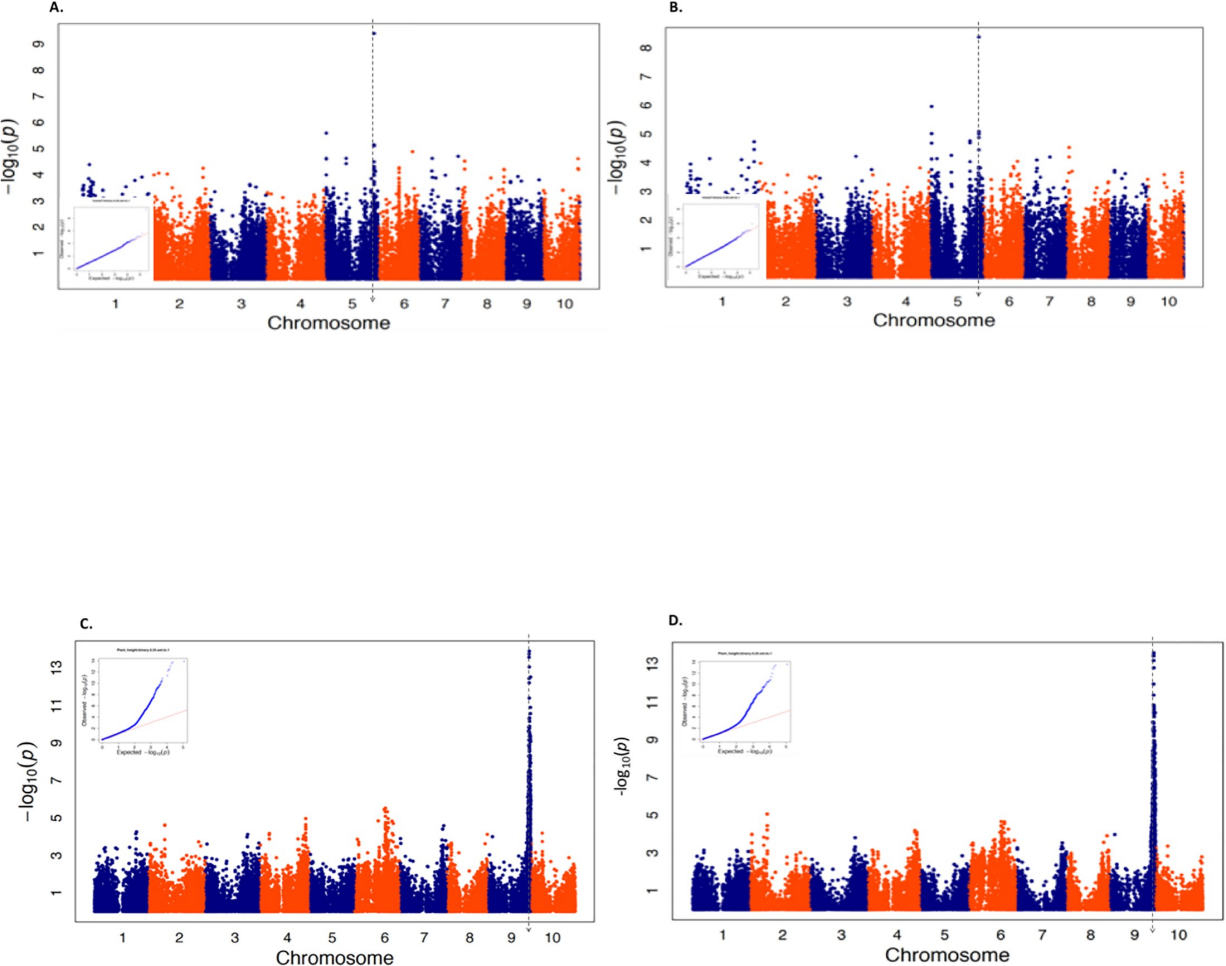
Results for two quantitative traits dichotomized at the 75^th^ percentile. Manhattan plots summarizing the genome-wide association study (GWAS) results for two quantitative traits dichotomized at the 75^th^ percentile. Each trait was analyzed using both the unified mixed linear model (MLM) and the logistic mixed model (LMM). Quantile quantile (QQ)-plots depicting the observed (Y-axis) and expected (X-axis) −log(10) *P*-values are inserted into each Manhattan plot. The Manhattan plots on each graph shows the physical bp position of each tested SNP in either the maize B73_RefGen v2 reference genome (for A and B) or the sorghum Btx623 v2.1 reference genome (for C and D) on the X-axis; while the −log(10) *P*-values from either the unified MLM (A and C) or LMM (B and D) on the Y-axis. The vertical line on each graph indicates the approximate location of the peak marker-trait associations identified in the previously-published GWAS of the original quantitative trait. (A) Results for a GWAS on dichotomized *α*-tocopherol measured in maize grain using the unified MLM. (B) Results for a GWAS on dichotomized *α*-tocopherol measured in maize grain using the LMM. (C) Results for a GWAS on dichotomized sorghum plant height using the unified MLM. (D) Results for a GWAS on dichotomized sorghum plant height using the LMM.

We then repeated the same analysis on the same three traits in maize and two traits in sorghum, this time with each trait dichotomized at the 75^th^ percentile. Similar to when these traits were dichotomized using the 50^th^ percentile, the LMM and the unified MLM appeared to sufficiently control for spurious associations (Table 2, Fig 2 and S9–S14 Figs). However, a different number of statistically significant associations were obtained from the unified MLM and the LMM when these traits were dichotomized using the 75^th^ percentile. Interestingly, the unified MLM identified four SNPs significantly associated with maize ear height at 10% FDR and one statistically significant marker-trait association for sorghum branch length at 5% FDR (Table 2 and S9 Fig). Despite these differences, the majority of the peak associated SNPs identified by the two dichotomizations of colocalized to the same genomic regions (Figs 1 and 2).

**Table 2:** Genome-wide association study results for quantitative traits that are dichotomized using the 75^th^ percentile. Number of statistically significant marker-trait associations and genomic control values from the unified mixed linear model and the logistic mixed model are presented.

Species	Dichotomized Trait	No. of Significant Associations				GC^a^ *λ* Values	
		5% FDR^b^		10% FDR			
		MLM^c^	LMM^d^	MLM	LMM	MLM	LMM
Maize	*α*-tocopherol	1	1	1	1	0.96	1.00
Maize	Zeaxanthin	0	0	0	0	1.05	1.01
Maize	Ear Height	0	0	4	0	1.02	1.05
Sorghum	Plant Height	593	495	756	642	1.08	1.06
Sorghum	Branch Length	1	0	1	0	1.02	0.95

^***a***^GC, Genomic control

^*b*^FDR, False discovery rate

^*c*^MLM, Unified mixed linear model

^*d*^LMM, Logistic mixed model

### Demonstration of superior control for spurious associations when using the LMM in the Goodman maize diversity panel

**Fig 3:**
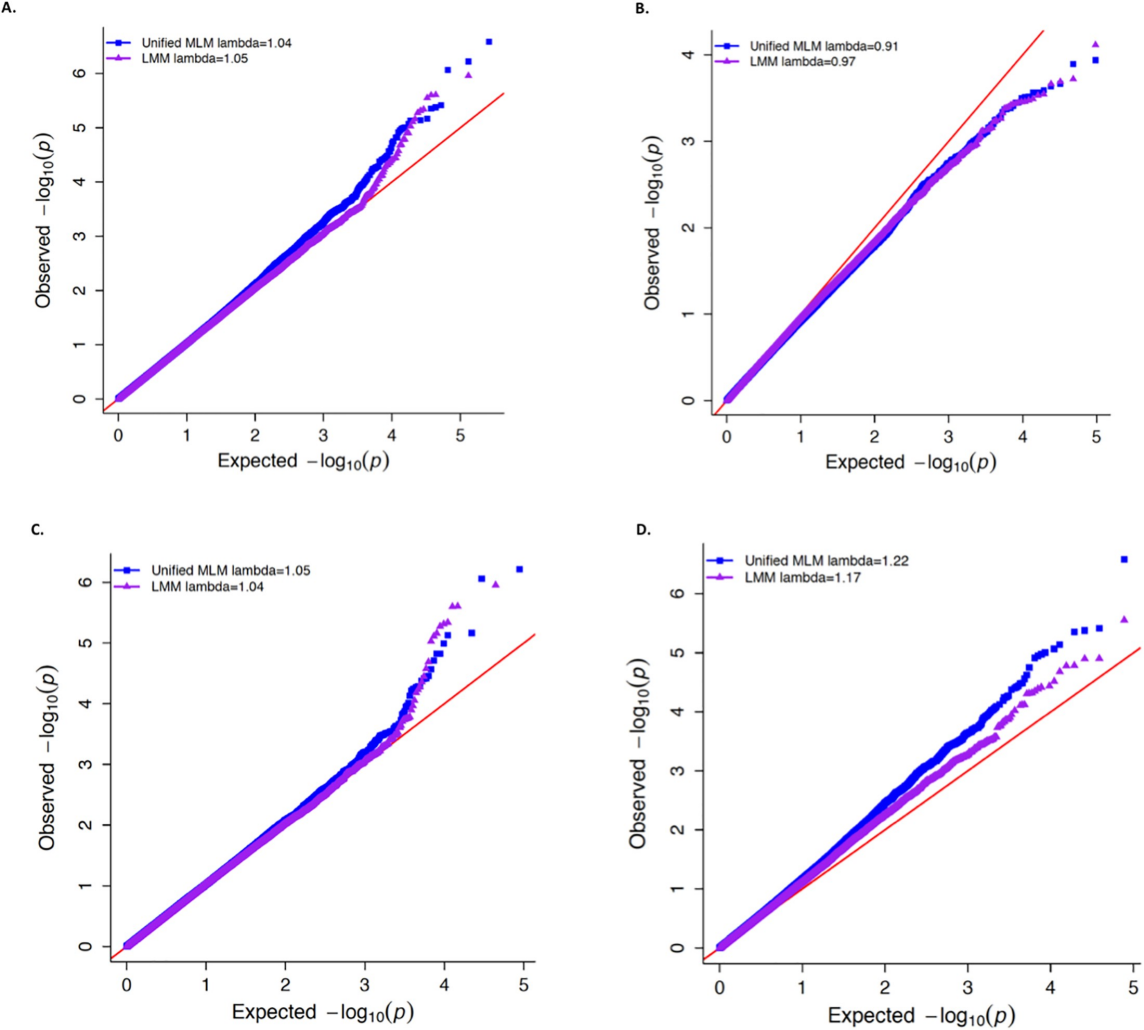
Distribution of -log10(P-values) for dichotomized maize ear height at the 75^th^ percentile. Quantile-quantile (QQ) plots showing the distribution of −log(10) *P*-values of 262,191 single nucleotide polymorphisms (SNPs) tested for association with dichotomized maize ear height at the 75^th^ percentile. On each plot the observed −log(10) *P*-values from the unified mixed linear model (MLM; blue squares) and logistic mixed model (LMM; purple triangles) are plotted against the expected −log(10) *P*-values. The value of lambda for genomic control for the unified MLM and LMM are presented in the legend of each plot. (A) QQ-plot for all 262,191 SNPs that are non-monomorphic within the tropical and non-tropical subpopulations of the Goodman diversity panel. (B) QQ-plot of the SNPs that were more common in the non-tropical subpopulation (i.e., the SNPs where the ratio of expected variance between tropical and non-tropical subpopulations was less than 0.80). (C) QQ-plot of SNPs that tended to have similar allele frequencies in both subpopulations (i.e., the SNPs where the ratio of expected variance between tropical and non-tropical lines were between 0.80 and 1.25). (D) QQ-plot of SNPs that were more common in the tropical subpopulation (i.e., the SNPs where the ratio of expected variance between tropical and non-tropical subpopulations was greater than 1.25).

One major pitfall of using the unified MLM to analyze binary traits is that it could inadequately control the type I error rate, especially when certain subpopulations have a substantially different proportion of “1’s” than the remaining subpopulations [8]. Here we demonstrate that it is possible for conditions that lead to such inadequate control to occur for a binary trait in agronomic data, and that the LMM provides superior control for spurious associations when used to analyze the same trait. The specific trait that we used to illustrate this point was ear height in the Goodman maize diversity panel dichotomized at the 75^th^ percentile. For this trait, a substantially greater proportion of tropical maize lines have “1’s” (0.48) compared to non-tropical lines (0.18), which results in a larger variance for this trait in the tropical subpopulation (Table 3). We observed that, for this trait, the unified MLM yielded noticeably inflated −log(10) *P*-values compared to those from the LMM when used to test SNPs that had more common allele frequencies in the tropical subpopulation (Fig 3). Although not as visually apparent, we also observed that for SNPs that were less common in the tropical subpopulation, the unified MLM produced −log(10) *P*-values that were more deflated than those produced by the LMM.

**Table 3:** Summary of observed values of maize ear height dichotomized using the 75^th^ percentile among the tropical and non-tropical subpopulations of 278 lines from Goodman maize diversity panel.

Subpopulation	No. Individuals	Proportion of “1’s” for Ear Height Dichotomized by 75^th^ percentile	Approximate variance of Dichotomized Ear Height^a^
Non-tropical	214	0.18	0.1476
Tropical	64	0.48	0.2496

^***a***^Variance is calculated by $\hat{\pi }\left(1-\hat{\pi }\right)$, where $\hat{\pi }$ is the observed proportion of individuals with “1’s” within a given subpopulation

**Fig 4:**
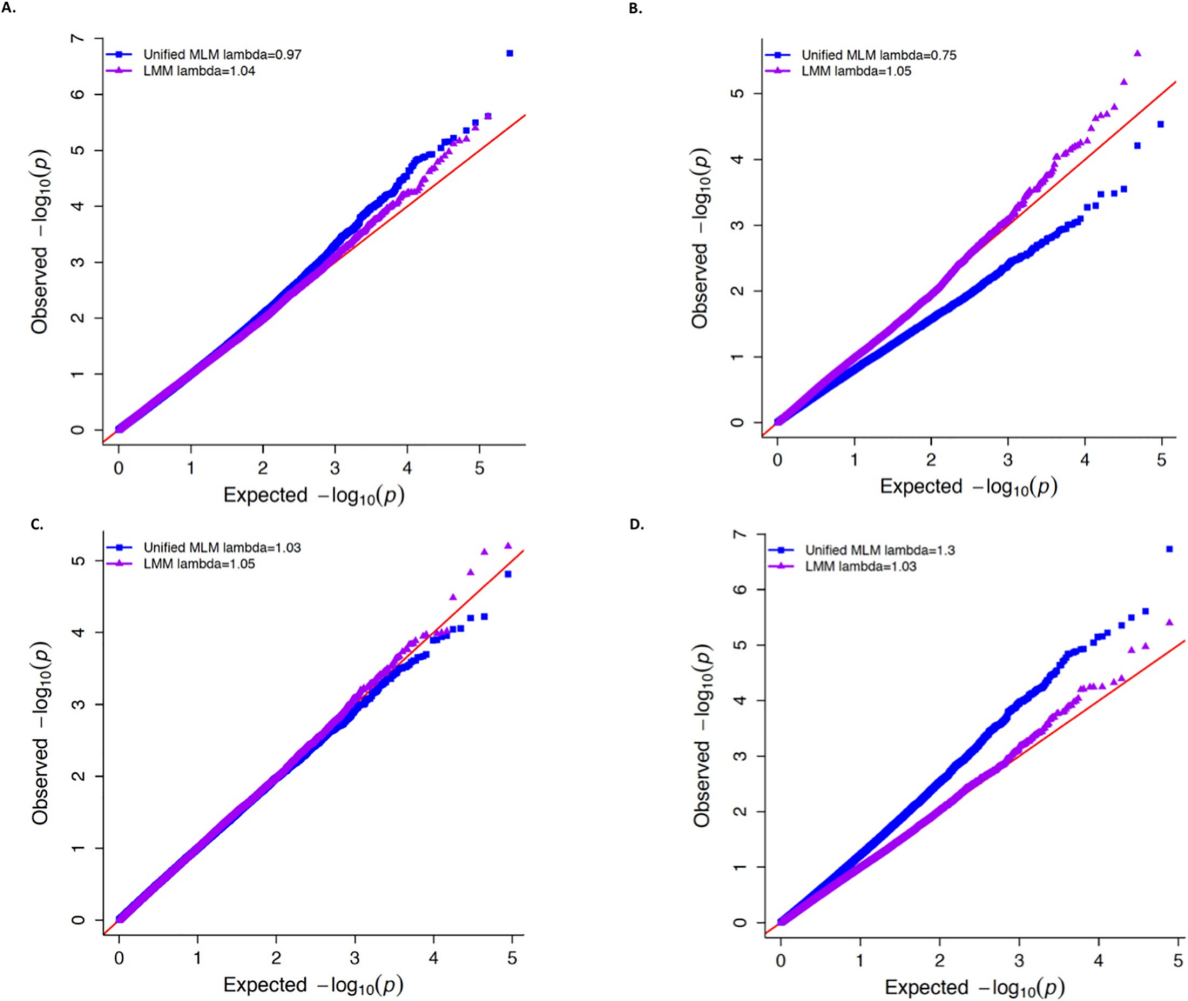
Distribution of -log10(P-values) for binary trait simulated in maize with unequal proportion of “1’s” in subpopulations. Quantile-quantile (QQ) plots showing the distribution of −log(10) *P*-values of 262,191 single nucleotide polymorphisms (SNPs) tested for association with binary trait where the probability of observing “1” differed between the tropical and non-tropical subpopulations. On each plot the observed −log(10) *P*-values from the unified mixed linear model (MLM; blue squares) and logistic mixed model (LMM; purple triangles) are plotted against the expected −log(10) *P*-values. The value of lambda for genomic control for the unified MLM and LMM are presented in the legend of each plot. (A) QQ-plot for all 262,191 SNPs that are non-monomorphic within the tropical and non-tropical subpopulations of the Goodman diversity panel. (B) QQ-plot of the SNPs that were more common in the non-tropical subpopulation (i.e., the SNPs where the ratio of expected variance between tropical and non-tropical subpopulations was less than 0.80). (C) QQ-plot of SNPs that tended to have similar allele frequencies in both subpopulations (i.e., the SNPs where the ratio of expected variance between tropical and non-tropical lines were between 0.80 and 1.25). (D) QQ-plot of SNPs that were more common in the tropical subpopulation (i.e., the SNPs where the ratio of expected variance between tropical and non-tropical subpopulations was greater than 1.25).

**Fig 5:**
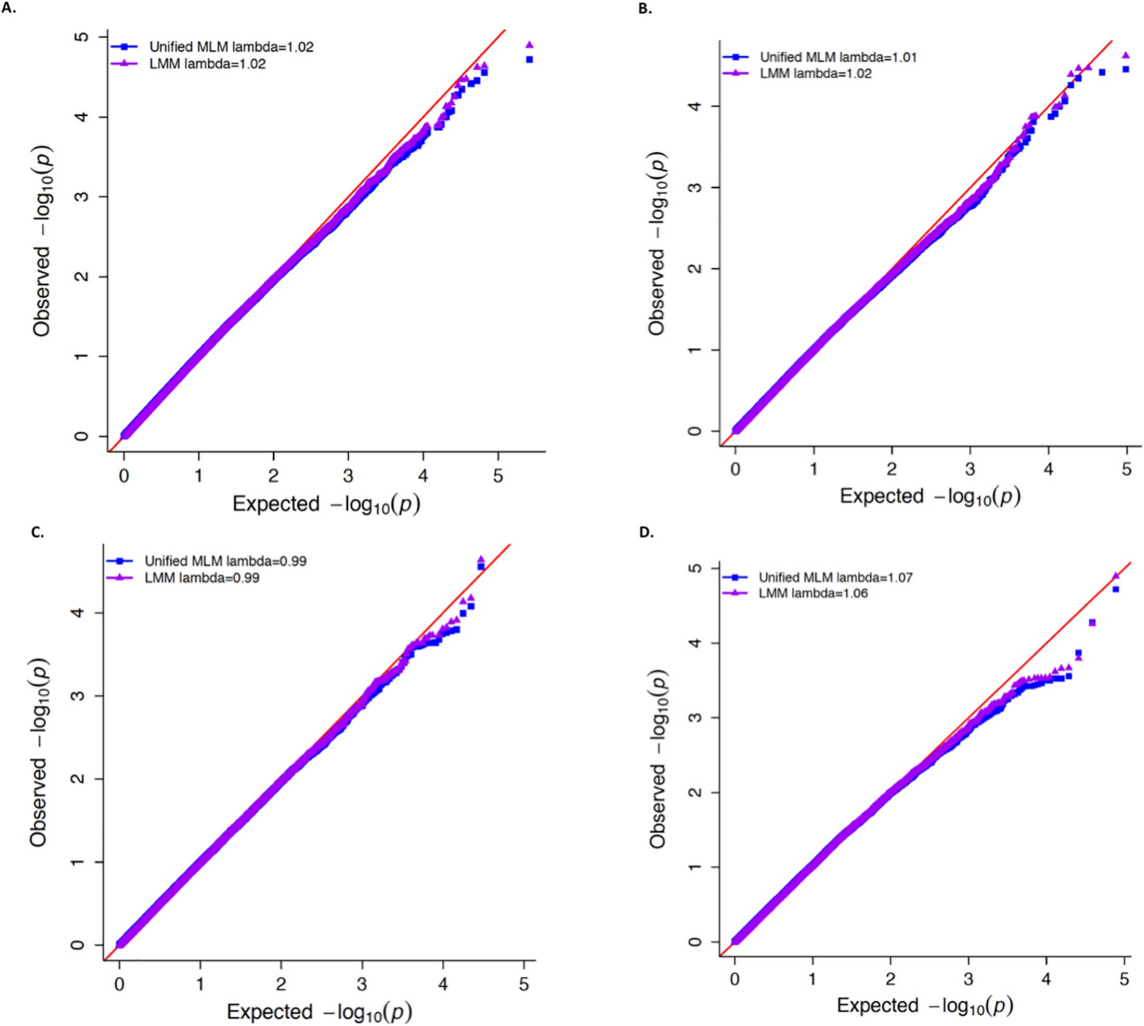
Distribution of -log10(P-values) for binary trait simulated in maize with equal proportion of “1’s” in subpopulations. Quantile-quantile (QQ) plots showing the distribution of −log(10) *P*-values of 262,191 single nucleotide polymorphisms (SNPs) tested for association with binary trait where the probability of observing “1” was the same between the tropical and non-tropical subpopulations. On each plot the observed −log(10) *P*-values from the unified mixed linear model (MLM; blue squares) and logistic mixed model (LMM; purple triangles) are plotted against the expected −log(10) *P*-values. The value of lambda for genomic control for the unified MLM and LMM are presented in the legend of each plot. (A) QQ-plot for all 262,191 SNPs that are non-monomorphic within the tropical and non-tropical subpopulations of the Goodman diversity panel. (B) QQ-plot of the SNPs that were more common in the non-tropical subpopulation (i.e., the SNPs where the ratio of expected variance between tropical and non-tropical subpopulations was less than 0.80). (C) QQ-plot of SNPs that tended to have similar allele frequencies in both subpopulations (i.e., the SNPs where the ratio of expected variance between tropical and non-tropical lines were between 0.80 and 1.25). (D) QQ-plot of SNPs that were more common in the tropical subpopulation (i.e., the SNPs where the ratio of expected variance between tropical and non-tropical subpopulations was greater than 1.25).

To put these results for dichotomized ear height into perspective, we also simulated two binary traits among the same 278 maize lines. One of these was simulated to have contrasting proportions of “1’s” in the tropical (*π* = 0.5) and non-tropical (*π* = 0.05) subpopulations, while the other was simulated to have an equal proportion of “1’s” (*π* = 0.5) in both subpopulations. Consequently, the former binary trait had substantially different variances in the two subpopulations, while the latter had equal variances regardless of subpopulation (Tables 4 and 5). Interestingly, the concordance between the *P*-values for the binary trait with equal proportion of “1’s” in both subpopulations was greater than those for the binary trait with unequal proportion of “1’s” in both subpopulations (S15 and S16 Figs). In any case, given that there were no genomic sources underlying the variability of these two simulated traits, the resulting distributions of *P*-values for testing *H*_0_: No marker-trait association from the LMM and unified MLM fitted at each marker were expected to adhere to the theoretical Uniform[0,1] distribution expected under any null hypothesis [46]. While Figs 4 and 5 show that the resulting distributions of *P*-values from the LMM reasonably follow this expected distribution, the *P*-values from the unified MLM deviate strongly from this theoretical distribution for the binary trait with unequal variances in the two subpopulations. This is particularly apparent for those SNPs that were less (Fig 4B) and more (Fig 4D) common in the tropical subpopulation. Thus, these findings demonstrate that it is possible for crop diversity panels to display the same properties described in [8] that lead to the unified MLM’s deficient control of type I errors when used to analyze binary traits.

**Table 4:** Summary the binary trait simulated on 278 lines from Goodman maize diversity panel where the probability of observing “1” differed in the tropical and subtropical subpopulations.

Subpopulation	No. Individuals	Values of *π* = *P*{*Binary trait* = 1} pulation	Variance of binary trait in each subpopulation^a^
Non-tropical	214	0.05	0.0475
Tropical	64	0.50	0.2500

^***a***^Variance is calculated by *π*(1 − *π*)

**Table 5:** Summary the binary trait simulated on 278 lines from Goodman maize diversity panel where the probability of observing “1” was the same in the tropical and subtropical subpopulations.

Subpopulation	No. Individuals	Values of *π* = *P*{*Binary trait* = 1} subpopulation	Variance of binary trait in each subpopulation^a^
Non-tropical	214	0.50	0.2500
Tropical	64	0.50	0.2500

^***a***^Variance is calculated by *π*(1 − *π*)

## Discussion

From a statistical perspective, the use of the unified MLM to analyze a binary trait is inappropriate because of violations in model assumptions. Using the computationally efficient implementation of GMMAT in the GENESIS R package [8,28] we dichotomized five agronomic traits in two crop diversity panels to explore the performance of a statistically appropriate analogue of the unified MLM for analyzing binary traits, namely the LMM. We demonstrated that the LMM was generally capable of detecting the same statistically significant marker-trait associations as those identified with the unified MLM; moreover these signals co-localized to the same genomic regions identified in the studies that presented the GWAS results of the original quantitative traits [31,38]. Finally, we conducted the analysis described in [8] using agronomic data to demonstrate through simulated and real phenotypes that the conditions leading to the unified MLM’s insufficient control of spurious associations can arise in crop diversity panels. Thus the work presented here provides an example of the usefulness and applicability of the LMM in diversity panels of two different crop species.

### Impact of species and proportion of “1’s” on LMM performance

To assess the robustness of the LMM’s performance to the crop species under study and the observed proportion of “1’s” for the studied binary trait, we performed our analyses in diversity panels from two separate species and dichotomized the studied quantitative traits based on two different percentiles. Our results suggest that both of these factors had minimal influence on the ability of our analyses to detect genomic signals that correspond to those identified in previous studies. While these findings are not particularly groundbreaking, they could be considered within the context of the genomic properties of the two species we analyzed. As an outcrosser, the average range of LD decay in maize [47] is substantially shorter than that of sorghum [38]. Thus regardless of the contrasting LD decay in these two species, the LMM was capable of yielding results that are consistent with previous analyses of the original quantitative trait. In a similar vein, despite that the expected variance of the binary traits dichotomized at the 75^th^ percentile were 25% less than those dichotomized at the 50^th^ percentile, the similarity between the genomic regions identified by the LMM as containing statistically significant signals between these two dichotomizations were generally similar (Figs 1 and 2). This suggest that for binary traits where the observed proportion of “1’s” resemble those that we considered, the proportion of “1’s” do not have an undue influence on the ability of the LMM to identify peak associations. Thus, it appears reasonable to conclude that for our study, the key factors driving the ability of the LMM to identify markers that are statistically significantly associated with these tested binary traits are the same genetic and non-genetic sources underlying the variability of the original quantitative traits.

### Demonstration of superior control for spurious associations of LMM

Using a dichotomized version of ear height and simulated binary traits in the Goodman maize diversity panel, we performed the procedure described in [8] to demonstrate that it is possible for the unified MLM to inadequately control for spurious associations when analyzing binary traits in crop diversity panels. Ubiquitously used in crop GWAS, diversity panels strive to encompass as wide a range of genetic diversity as possible [1] and this typically translates to the presence of subpopulation structure among the individuals comprising a panel. Thus when the unified MLM is fitted to binary traits that are highly correlated with population structure, the resulting residuals could theoretically be heteroscedastic. The use of the unified MLM on such a binary trait would consequently be especially prone to inadequate control of spurious associations in a manner similar to what was demonstrated in [8] and in this work. Although such insufficient control by the unified MLM is likely due to heteroscedasticity of the residuals and other violations of model assumptions instead of any unique characteristic of crop diversity panels, it is nevertheless important to demonstrate these properties for crop data. In this light, implementation of the LMM for the GWAS of binary agronomical traits would add to the efforts already made to control for sources of false positive marker-trait associations [4,48,49] that frequently occur in diversity panels.

### The use of the LMM for GWAS in binary and binomial agronomical traits

Prior to the implementation of GMMAT in the GENESIS R package, the inherent computational burden associated with fitting the LMM would have rendered its use in a GWAS of hundreds of thousands of SNPs impractical. As such, this implementation has potential to significantly benefit the agronomical GWAS research community because it enables the use of arguably the most statistically appropriate model for analyzing binary traits on a genome-wide scale. To make this implementation even more relevant to this community, we suggest that the current implementation of GMMAT be augmented with the ability to analyze binomially distributed traits. Because a typical experimental unit in data sets obtained from crops grown in the field is a plot consisting of at least several plants, traits such as the number of plants that experience stalk lodging can theoretically be well-approximated with a binomial distribution. Hence, such an extension to the current implementation of GMMAT could eliminate the need for agronomical researchers to incorrectly use the unified MLM to analyze this class of non-normally distributed traits.

### Conclusion

It is imperative the most statistically appropriate models are used to analyze binary traits. Until recently, it was computationally infeasible to implement such a model for the GWAS of binary traits in crop diversity panels, namely the LMM. Due to the implementation of GMMAT in the GENESIS R package, it is now practical to use this model for such an analysis. We hope that the simple study presented here shows the usefulness of this approach for analyzing binary traits in a crop diversity panel, and we therefore advocate its use for GWAS among the crop quantitative genetics community.

## Supporting information

S1 Table An assessment of computational time for performing a genome-wide association study for marker sets of various sizes using the unified mixed linear model and the logistic mixed model. All analyses were performed on a MacBook Pro laptop.
(DOCX)

S1 Fig Scree plot from a principal component analysis of genome-wide markers in the Goodman maize diversity panel. The X-axis is the principal component number and the Y-axis is the amount of variance explained. This plot suggests that the first three principal components adequately explain the variation among the genome-wide markers.
(TIFF)

S2 Fig Scree plot from a principal component analysis of genome-wide markers in the US sorghum association panel. The X-axis is the principal component number and the Y-axis is the amount of variance explained. This plot suggests that the first three principal components adequately explain the variation among the genome-wide markers.
(TIFF)

S3 Fig Manhattan plots summarizing the genome-wide association study (GWAS) results for all analyzed quantitative traits dichotomized at the 50^th^ percentile. The specific trait and species of each plot is indicated in the row labels. The X-axis of each graph is physical position of either the B73_RefGen v2 position of the maize genome (for first three rows) or the Btx623 v2.1 position of the sorghum genome (for the bottom two rows), and the Y-axis shows the −log(10) *P*-values from either the unified mixed linear model (MLM; presented in the left column) or the logistic mixed model (LMM; presented in the right column). Quantile quantile (QQ)-plots depicting the observed (Y-axis) and expected (X-axis) −log(10) *P*-values are inserted into each Manhattan plot.
(TIFF)

S4 Fig Comparison of -log10(*P*-values) from the logistic mixed model and the unified mixed linear model. Plot of -log10(*P*-values) of SNPs from the logistic mixed model (Y-axis) against those from the unified mixed linear model (X-axis) for the genome-wide association study conducted for *α*-tocopherol levels in maize grain in the Goodman diversity panel dichotomized at the 50^th^ percentile. Both sets of -log10(*P*-values) are from testing *H*_0_: no association between the tested SNP and the phenotype.
(TIFF)

S5 Fig Comparison of -log10(*P*-values) from the logistic mixed model and the unified mixed linear model. Plot of -log10(*P*-values) of SNPs from the logistic mixed model (Y-axis) against those from the unified mixed linear model (X-axis) for the genome-wide association study conducted for zeaxanthin levels in maize grain in the Goodman diversity panel dichotomized at the 50^th^ percentile. Both sets of -log10(*P*-values) are from testing *H*_0_: no association between the tested SNP and the phenotype.
(TIFF)

S6 Fig Comparison of -log10(*P*-values) from the logistic mixed model and the unified mixed linear model. Plot of -log10(*P*-values) of SNPs from the logistic mixed model (Y-axis) against those from the unified mixed linear model (X-axis) for the genome-wide association study conducted for maize ear height in the Goodman diversity panel dichotomized at the 50^th^ percentile. Both sets of -log10(*P*-values) are from testing *H*_0_: no association between the tested SNP and the phenotype.
(TIFF)

S7 Fig Comparison of -log10(*P*-values) from the logistic mixed model and the unified mixed linear model. Plot of -log10(*P*-values) of SNPs from the logistic mixed model (Y-axis) against those from the unified mixed linear model (X-axis) for the genome-wide association study conducted for sorghum plant height in the US sorghum association panel dichotomized at the 50^th^ percentile. Both sets of -log10(*P*-values) are from testing *H*_0_: no association between the tested SNP and the phenotype.
(TIFF)

S8 Fig Comparison of -log10(*P*-values) from the logistic mixed model and the unified mixed linear model. Plot of -log10(*P*-values) of SNPs from the logistic mixed model (Y-axis) against those from the unified mixed linear model (X-axis) for the genome-wide association study conducted for sorghum branch length in the US sorghum association panel dichotomized at the 50^th^ percentile. Both sets of -log10(*P*-values) are from testing *H*_0_: no association between the tested SNP and the phenotype.
(TIFF)

S9 Fig Manhattan plots summarizing the genome-wide association study (GWAS) results for all analyzed quantitative traits dichotomized at the 75^th^ percentile. The specific trait and species of each plot is indicated in the row labels. The X-axis of each graph is physical position of either the B73_RefGen v2 position of the maize genome (for first three rows) or the Btx623 v2.1 position of the sorghum genome (for the bottom two rows), and the Y-axis shows the −log(10) *P*-values from either the unified mixed linear model (MLM; presented in the left column) or the logistic mixed model (LMM; presented in the right column). Quantile quantile (QQ)-plots depicting the observed (Y-axis) and expected (X-axis) −log(10) *P*-values are inserted into each Manhattan plot.
(TIFF)

S10 Fig Comparison of -log10(*P*-values) from the logistic mixed model and the unified mixed linear model. Plot of -log10(*P*-values) of SNPs from the logistic mixed model (Y-axis) against those from the unified mixed linear model (X-axis) for the genome-wide association study conducted for *α*-tocopherol levels in maize grain in the Goodman diversity panel dichotomized at the 75^th^ percentile. Both sets of -log10(*P*-values) are from testing *H*_0_: no association between the tested SNP and the phenotype.
(TIFF)

S11 Fig Comparison of -log10(*P*-values) from the logistic mixed model and the unified mixed linear model. Plot of -log10(*P*-values) of SNPs from the logistic mixed model (Y-axis) against those from the unified mixed linear model (X-axis) for the genome-wide association study conducted for zeaxanthin levels in maize grain in the Goodman diversity panel dichotomized at the 75^th^ percentile. Both sets of -log10(*P*-values) are from testing *H*_0_: no association between the tested SNP and the phenotype.
(TIFF)

S12 Fig Comparison of -log10(*P*-values) from the logistic mixed model and the unified mixed linear model. Plot of -log10(*P*-values) of SNPs from the logistic mixed model (Y-axis) against those from the unified mixed linear model (X-axis) for the genome-wide association study conducted for maize ear height in the Goodman diversity panel dichotomized at the 75^th^ percentile. Both sets of -log10(*P*-values) are from testing *H*_0_: no association between the tested SNP and the phenotype.
(TIFF)

S13 Fig Comparison of -log10(*P*-values) from the logistic mixed model and the unified mixed linear model. Plot of -log10(*P*-values) of SNPs from the logistic mixed model (Y-axis) against those from the unified mixed linear model (X-axis) for the genome-wide association study conducted for sorghum plant height in the US sorghum association panel dichotomized at the 75^th^ percentile. Both sets of -log10(*P*-values) are from testing *H*_0_: no association between the tested SNP and the phenotype.
(TIFF)

S14 Fig Comparison of -log10(*P*-values) from the logistic mixed model and the unified mixed linear model. Plot of -log10(*P*-values) of SNPs from the logistic mixed model (Y-axis) against those from the unified mixed linear model (X-axis) for the genome-wide association study conducted for sorghum branch length in the US sorghum association panel dichotomized at the 75^th^ percentile. Both sets of -log10(*P*-values) are from testing *H*_0_: no association between the tested SNP and the phenotype.
(TIFF)

S15 Fig Comparison of -log10(*P*-values) from the logistic mixed model and the unified mixed linear model. Plot of -log10(*P*-values) of SNPs from the logistic mixed model (Y-axis) against those from the unified mixed linear model (X-axis) for the genome-wide association study conducted for binary trait *Y* simulated in the Goodman maize diversity panel where *P*{*Y* = 1} = 0.5 in the non-tropical subpopulation and *P*{*Y* = 1} = 0.05 in the non-tropical subpopulation. Both sets of -log10(*P*-values) are from testing *H*_0_: no association between the tested SNP and the phenotype.
(TIFF)

S16 Fig Comparison of -log10(*P*-values) from the logistic mixed model and the unified mixed linear model. Plot of -log10(*P*-values) of SNPs from the logistic mixed model (Y-axis) against those from the unified mixed linear model (X-axis) for the genome-wide association study conducted for binary trait *Y* simulated in the Goodman maize diversity panel where the *P*{*Y* = 1} = 0.5 regardless of the subpopulation. Both sets of -log10(*P*-values) are from testing *H*_0_: no association between the tested SNP and the phenotype.
(TIFF)
