# Supplementary material for: An assessment of the performance of the logistic mixed model for analyzing binary traits in maize and sorghum diversity panels
Source: PLoS One. 2018 Nov 21;13(11):e0207752. doi: 10.1371/journal.pone.0207752 (PMC6248992; doi:10.1371/journal.pone.0207752)
Supplement: S1 Table — All analyses were performed on a MacBook Pro laptop. (DOCX) [file pone.0207752.s001.docx]

**S1 Table.** An assessment of computational time for performing a genome-wide association study for marker sets of various sizes using the unified mixed linear model and the logistic mixed model. All analyses were performed on a MacBook Pro laptop.

| Species | No. Individuals |  | Number of markers tested*^a^* | | | Trait | |  | Time to conduct MLM*^b^*-based GWAS*^c^*  (seconds) | | | Time to conduct LMM-based GWAS (seconds) | | |  |  |
| --- | --- | --- | --- | --- | --- | --- | --- | --- | --- | --- | --- | --- | --- | --- | --- | --- |
| Maize | 278 |  | 2,553 |  |  | | Ear Height dichotomized at the 50^th^ percentile | | |  | 23.75 | | 6.67 | | |  |
| Maize | 278 |  | 49,491 |  |  | | Ear Height dichotomized at the 50^th^ percentile | | |  | 130.70 | | | 57.78 | | |
| Maize | 278 |  | 247,679*^e^* |  |  | | Ear Height dichotomized at the 50^th^ percentile | | |  | 291.09 | | | 64.08 | | |
| Sorghum | 320 |  | 115,167 |  |  | | Plant Height dichotomized at the 50^th^ percentile | | |  | 193.72 | | | 128.00 | | |

***^a^***Descriptions of these markers are provided in the Materials and Methods

*^b^*MLM, Unified mixed linear model

*^c^*GWAS, Genome-wide association study

*^d^*LMM, Logistic mixed model

*^e^*The input files for these markers were subdivided into ten chromosomes. When GWAS was ran using the unified MLM in in GAPIT, each marker file was read in separately during the analysis. When GWAS was ran on the LMM in GENESIS, all markers were already read into the R workspace.
