# Supplementary figures and images for: An assessment of the performance of the logistic mixed model for analyzing binary traits in maize and sorghum diversity panels
Source: PLoS One. 2018 Nov 21;13(11):e0207752. doi: 10.1371/journal.pone.0207752 (PMC6248992; doi:10.1371/journal.pone.0207752)

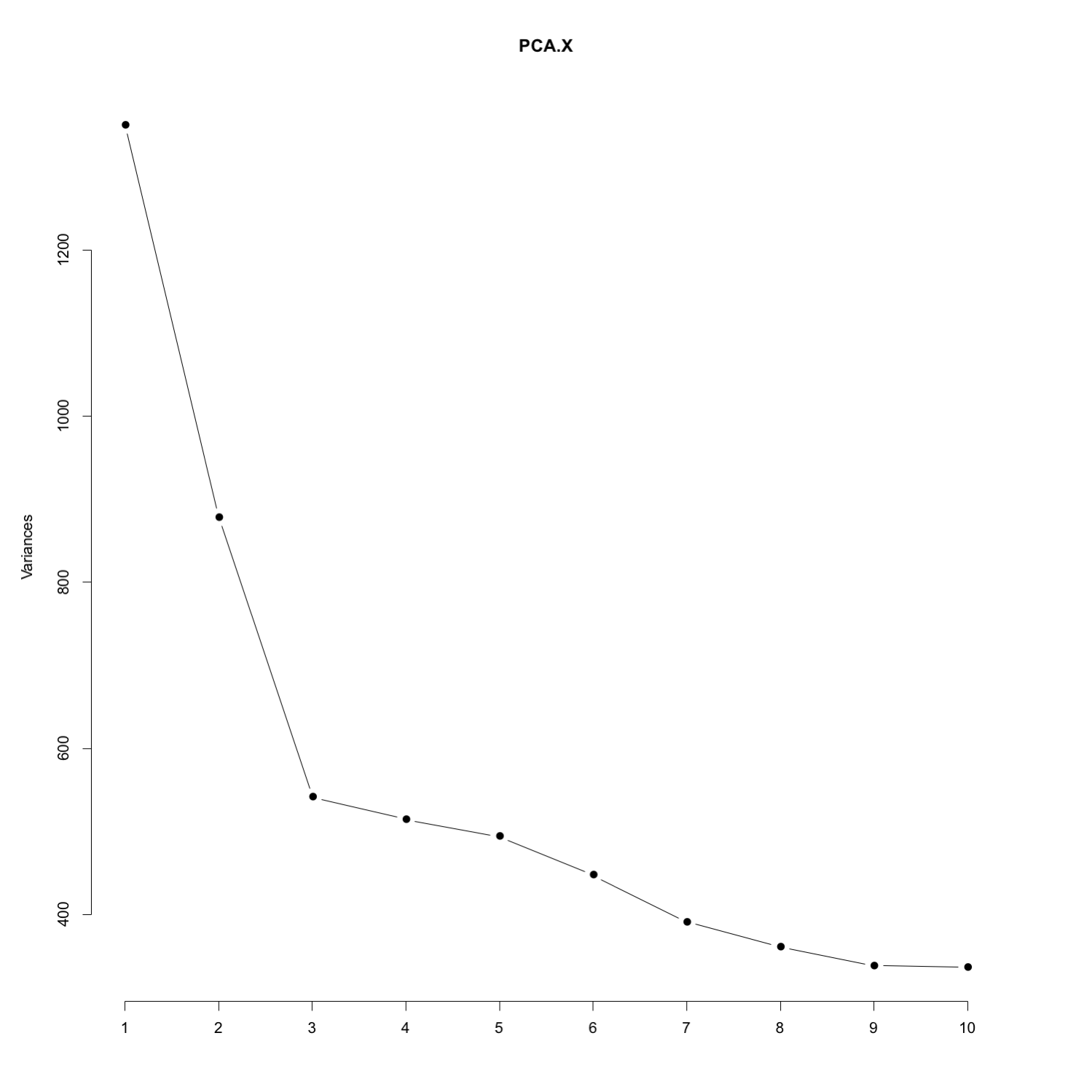

Supplement: S1 Fig — The X-axis is the principal component number and the Y-axis is the amount of variance explained. This plot suggests that the first three principal components adequately explain the variation among the genome-wide markers. (TIFF) [file pone.0207752.s002.tiff]

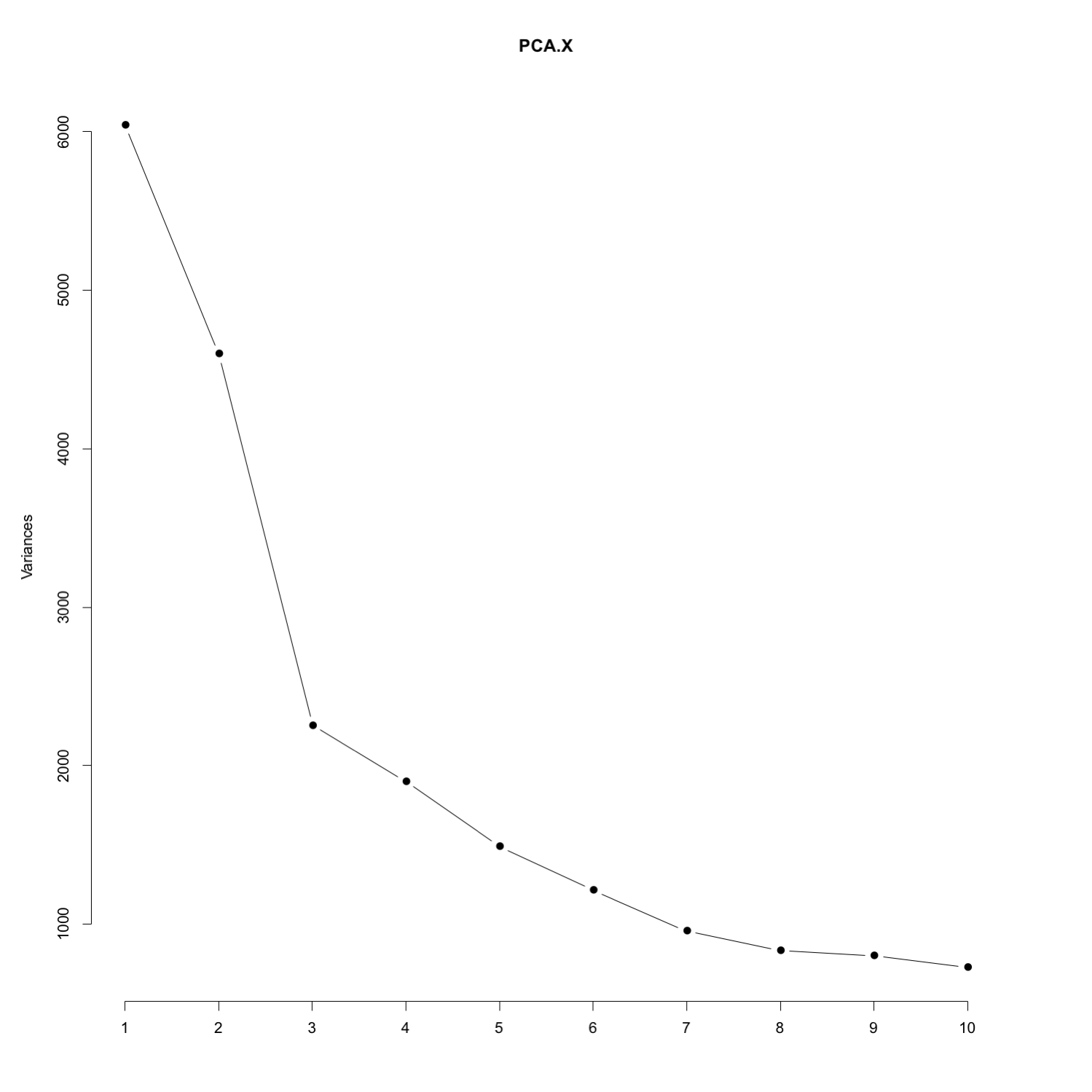

Supplement: S2 Fig — The X-axis is the principal component number and the Y-axis is the amount of variance explained. This plot suggests that the first three principal components adequately explain the variation among the genome-wide markers. (TIFF) [file pone.0207752.s003.tiff]

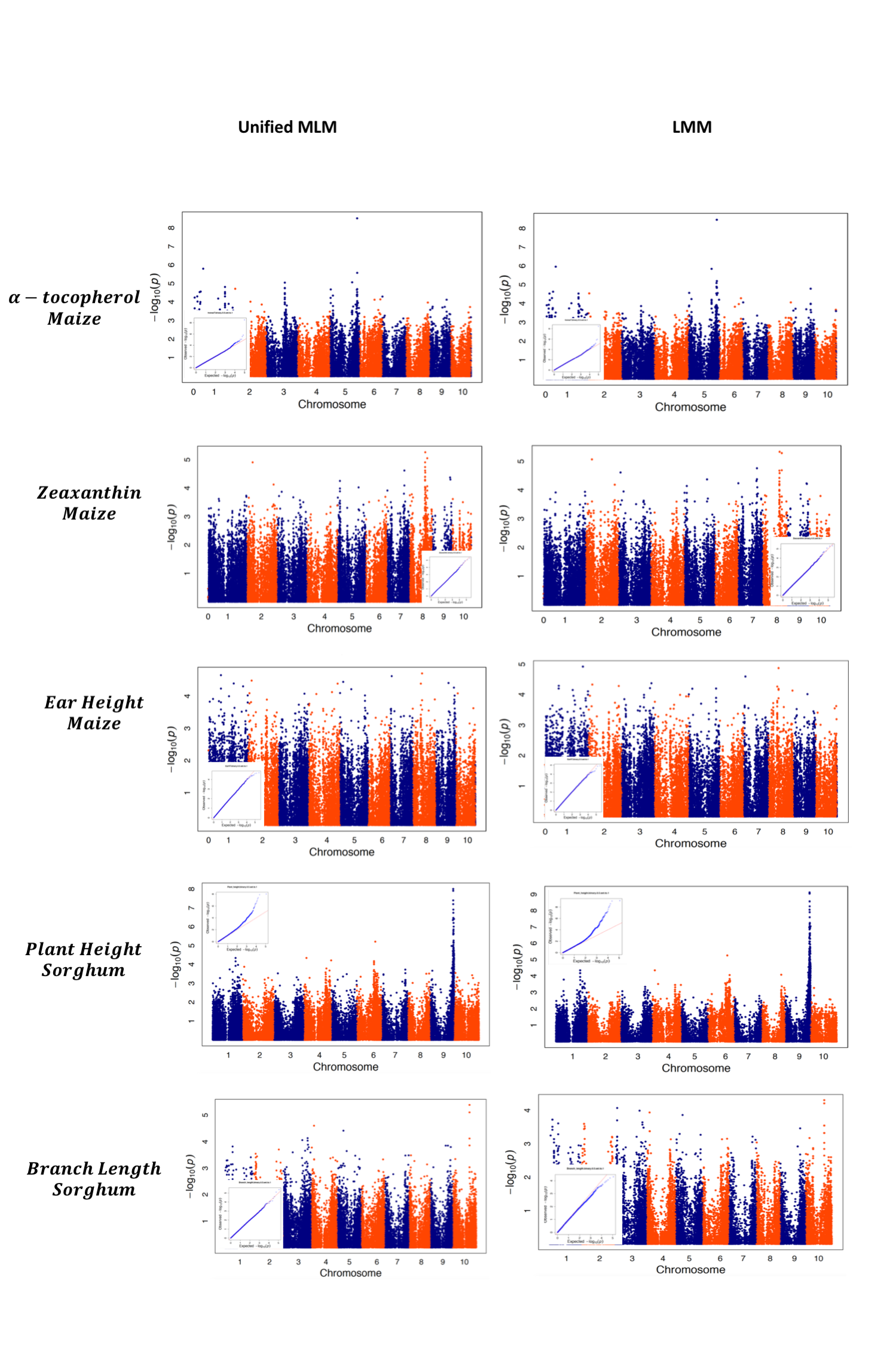

Supplement: S3 Fig — The specific trait and species of each plot is indicated in the row labels. The X-axis of each graph is physical position of either the B73_RefGen v2 position of the maize genome (for first three rows) or the Btx623 v2.1 position of the sorghum genome (for the bottom two rows), and the Y-axis shows the −log(10) P-values from either the unified mixed linear model (MLM; presented in the left column) or the logistic mixed model (LMM; presented in the right column). Quantile quantile (QQ)-plots depicting the observed (Y-axis) and expected (X-axis) −log(10) P-values are inserted into each Manhattan plot. (TIFF) [file pone.0207752.s004.tiff]

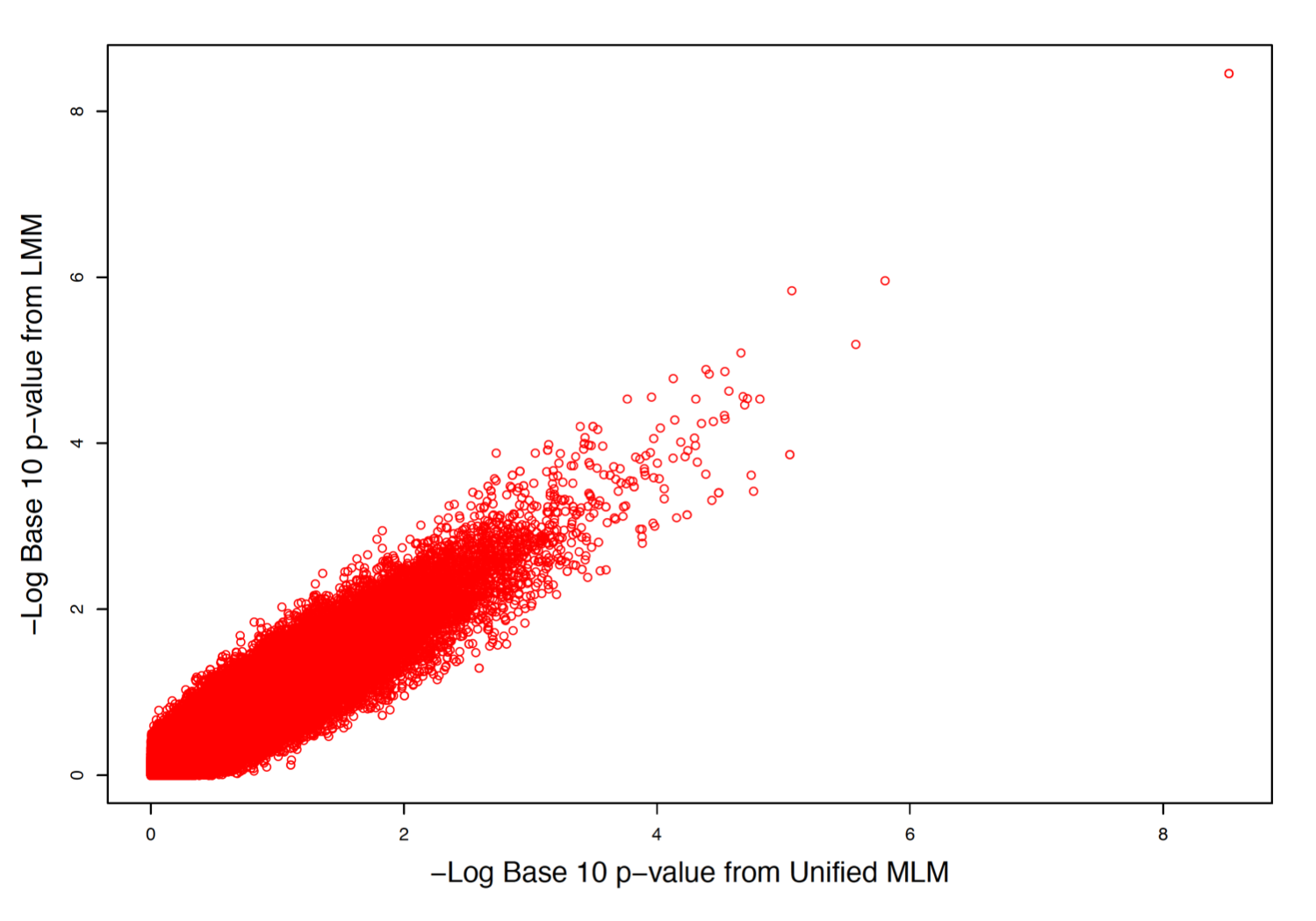

Supplement: S4 Fig — Plot of -log10(P-values) of SNPs from the logistic mixed model (Y-axis) against those from the unified mixed linear model (X-axis) for the genome-wide association study conducted for α-tocopherol levels in maize grain in the Goodman diversity panel dichotomized at the 50th percentile. Both sets of -log10(P-values) are from testing H0: no association between the tested SNP and the phenotype. (TIFF) [file pone.0207752.s005.tiff]

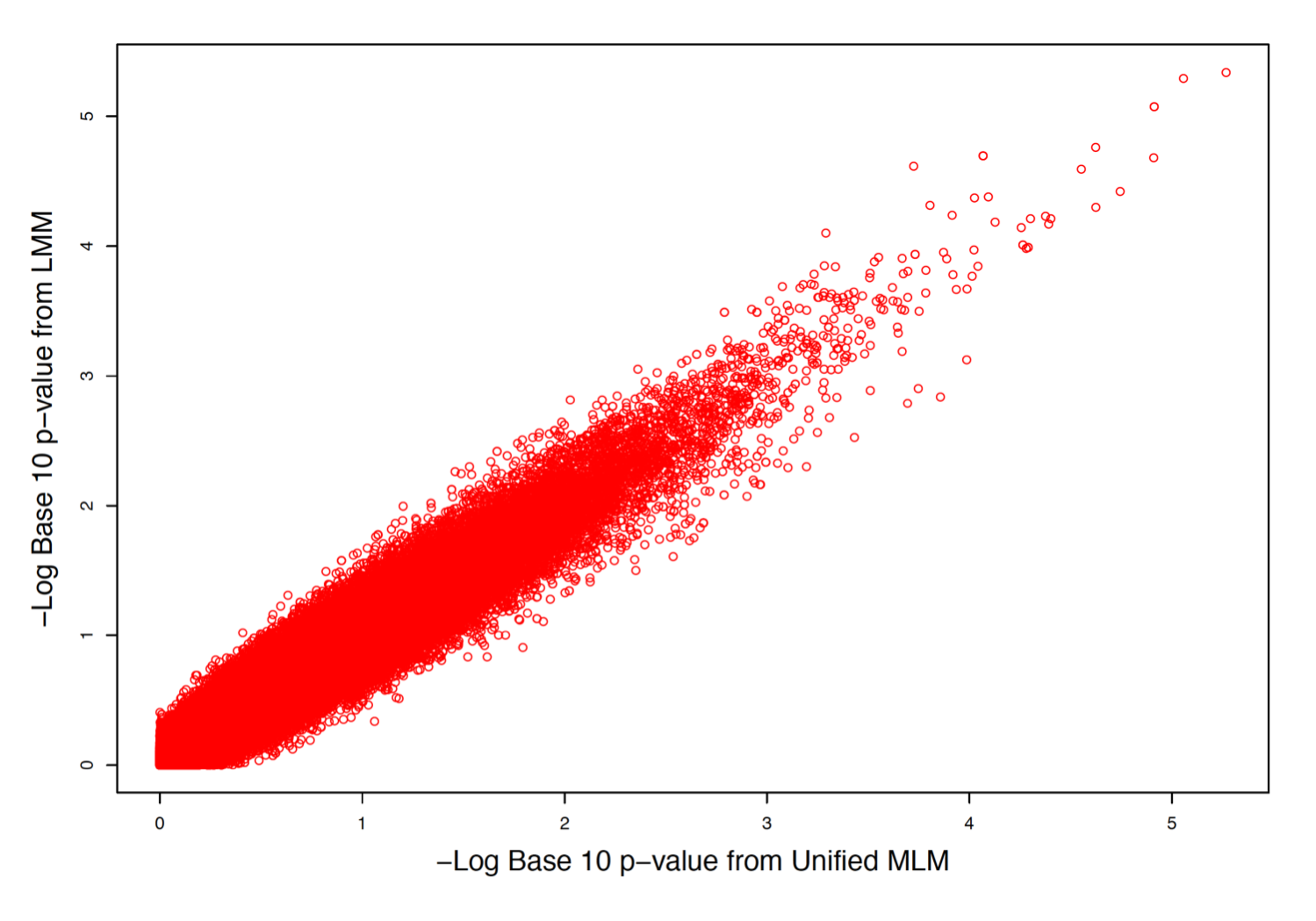

Supplement: S5 Fig — Plot of -log10(P-values) of SNPs from the logistic mixed model (Y-axis) against those from the unified mixed linear model (X-axis) for the genome-wide association study conducted for zeaxanthin levels in maize grain in the Goodman diversity panel dichotomized at the 50th percentile. Both sets of -log10(P-values) are from testing H0: no association between the tested SNP and the phenotype. (TIFF) [file pone.0207752.s006.tiff]

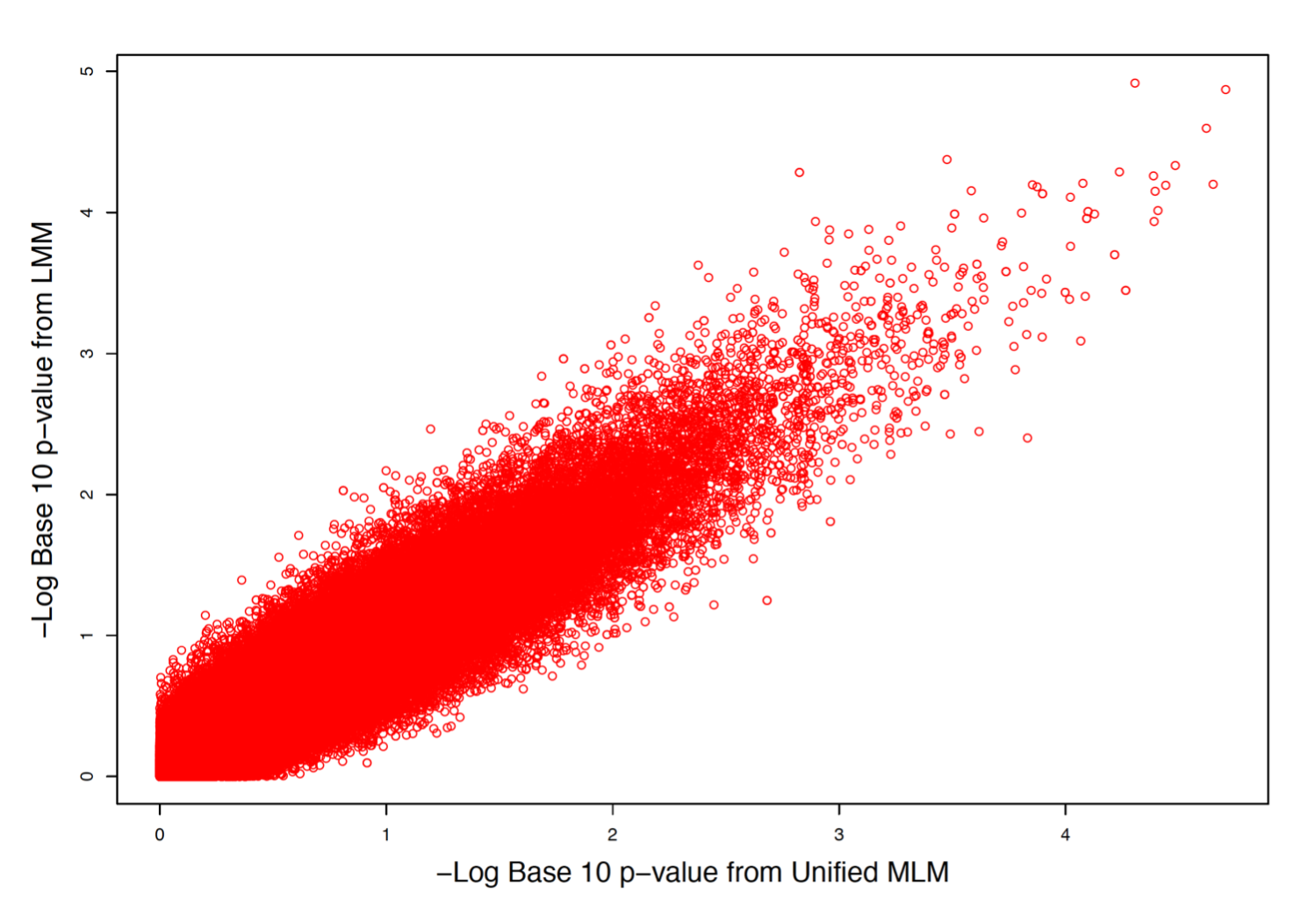

Supplement: S6 Fig — Plot of -log10(P-values) of SNPs from the logistic mixed model (Y-axis) against those from the unified mixed linear model (X-axis) for the genome-wide association study conducted for maize ear height in the Goodman diversity panel dichotomized at the 50th percentile. Both sets of -log10(P-values) are from testing H0: no association between the tested SNP and the phenotype. (TIFF) [file pone.0207752.s007.tiff]

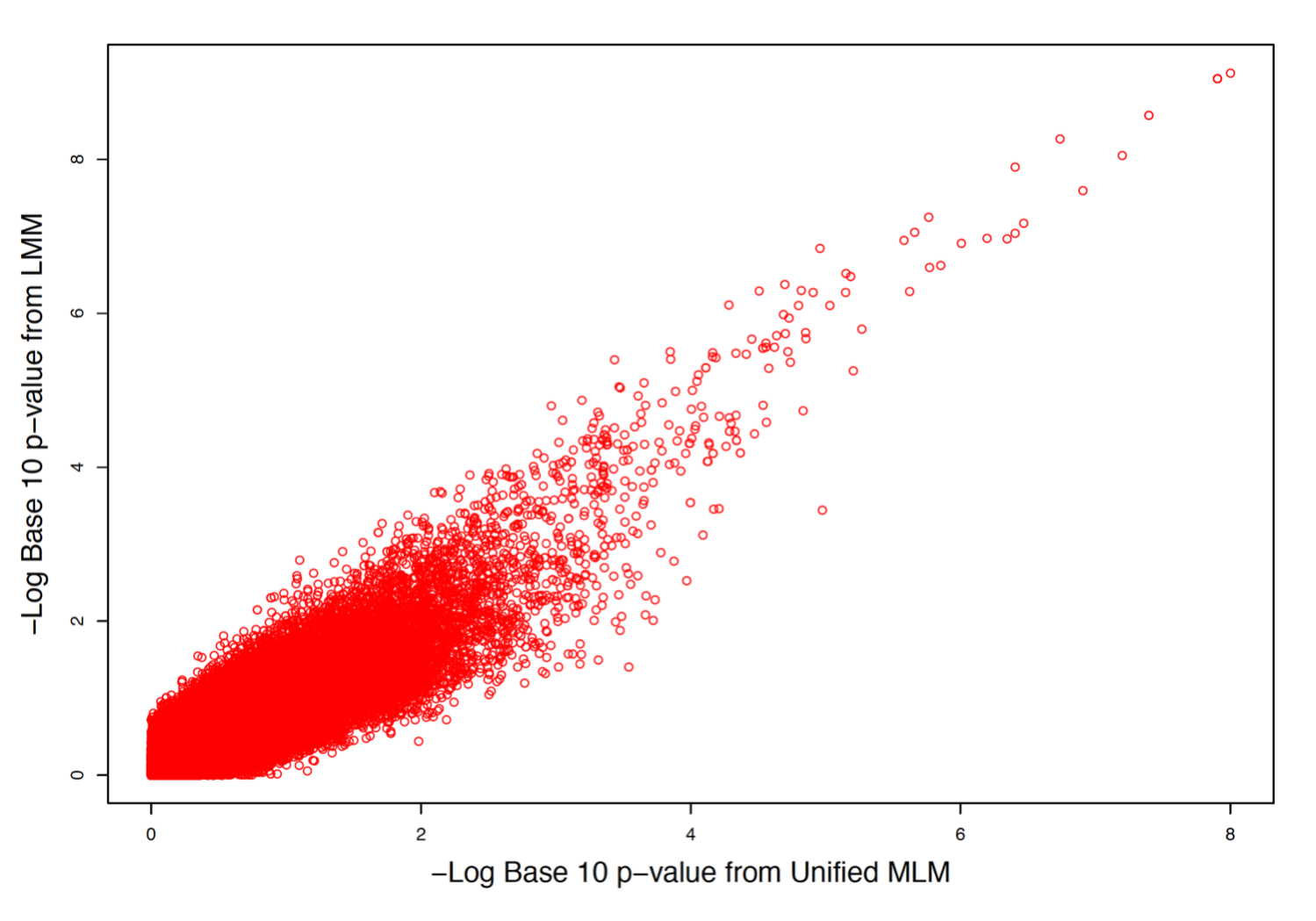

Supplement: S7 Fig — Plot of -log10(P-values) of SNPs from the logistic mixed model (Y-axis) against those from the unified mixed linear model (X-axis) for the genome-wide association study conducted for sorghum plant height in the US sorghum association panel dichotomized at the 50th percentile. Both sets of -log10(P-values) are from testing H0: no association between the tested SNP and the phenotype. (TIFF) [file pone.0207752.s008.tiff]

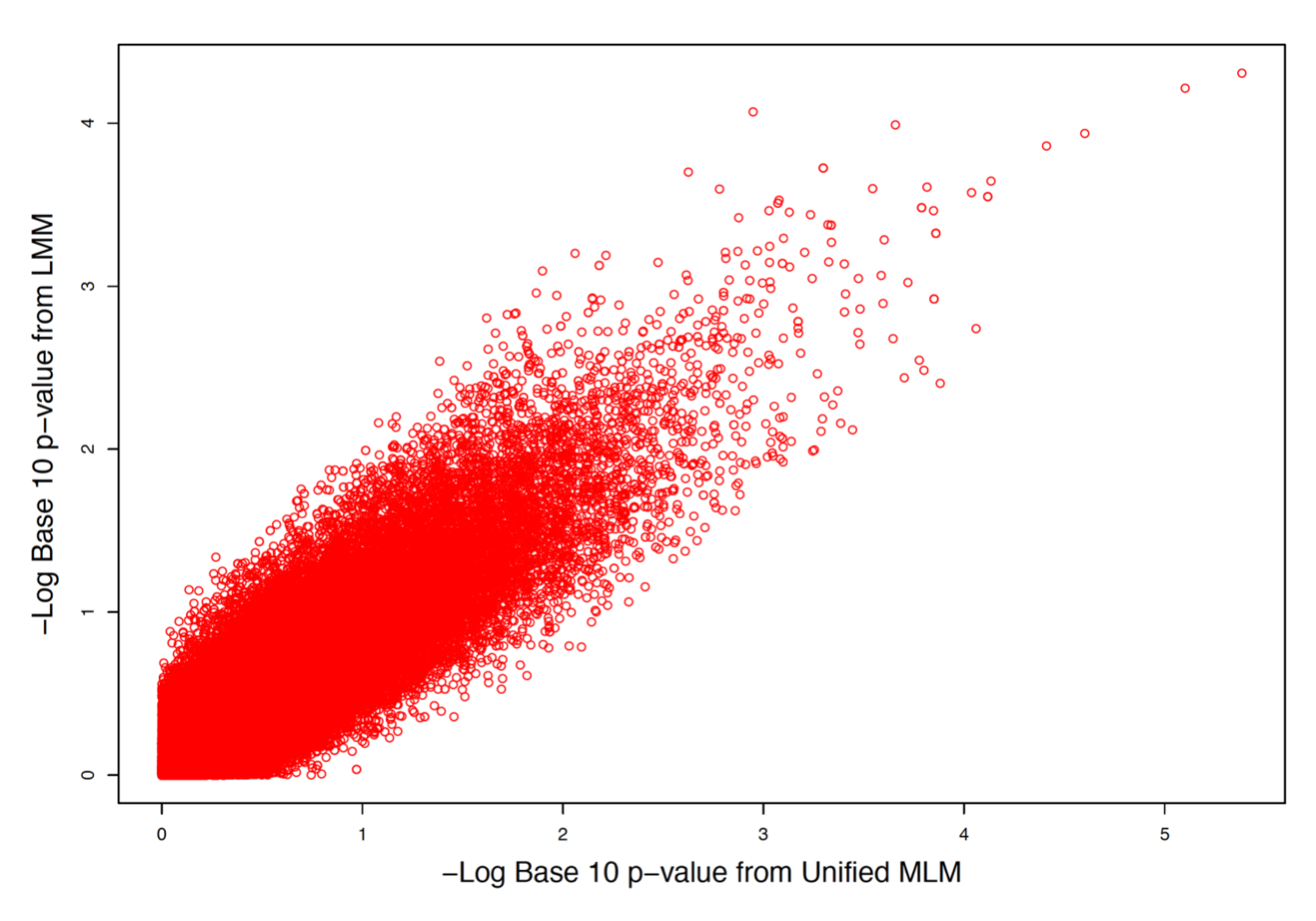

Supplement: S8 Fig — Plot of -log10(P-values) of SNPs from the logistic mixed model (Y-axis) against those from the unified mixed linear model (X-axis) for the genome-wide association study conducted for sorghum branch length in the US sorghum association panel dichotomized at the 50th percentile. Both sets of -log10(P-values) are from testing H0: no association between the tested SNP and the phenotype. (TIFF) [file pone.0207752.s009.tiff]

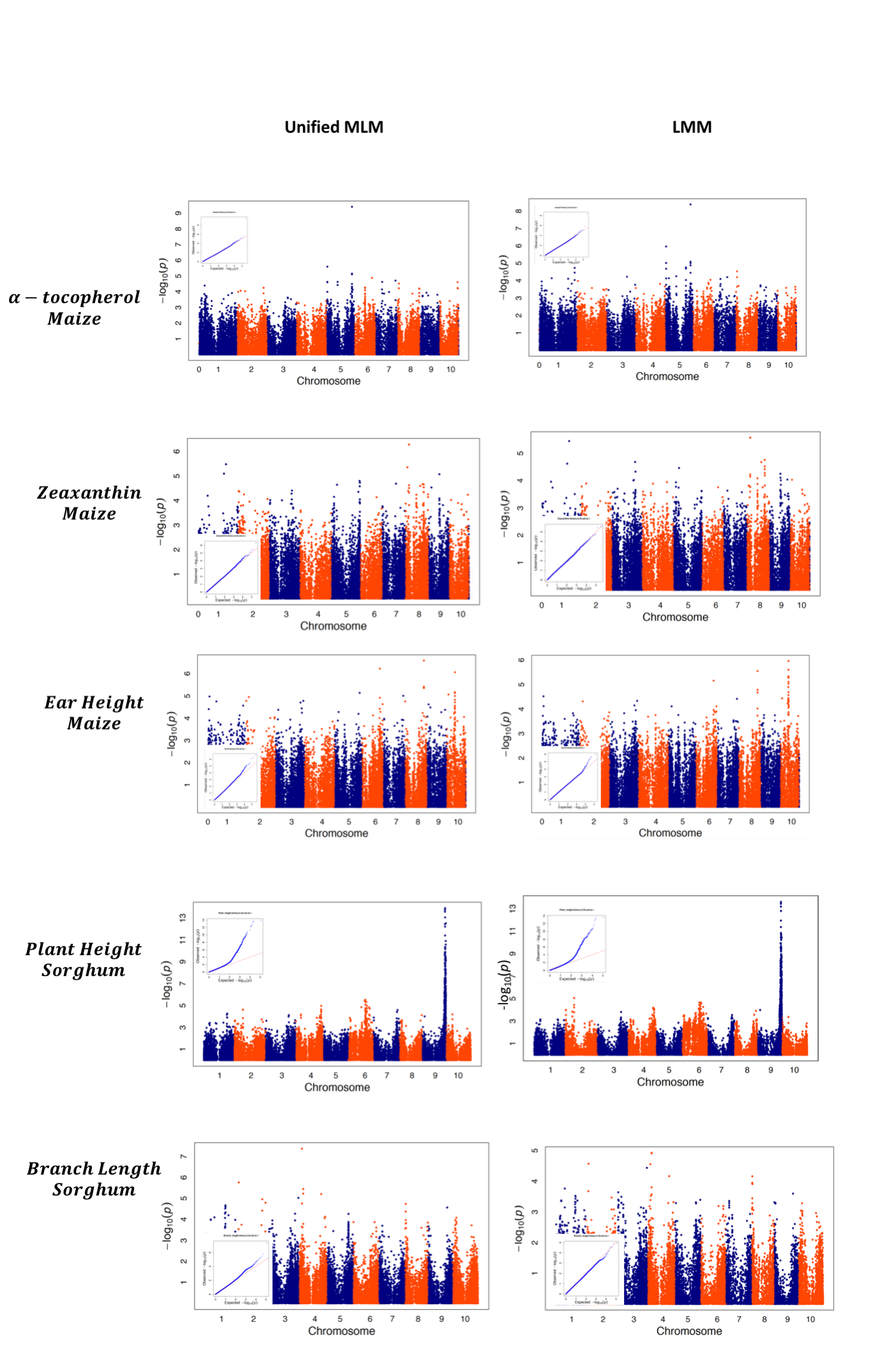

Supplement: S9 Fig — The specific trait and species of each plot is indicated in the row labels. The X-axis of each graph is physical position of either the B73_RefGen v2 position of the maize genome (for first three rows) or the Btx623 v2.1 position of the sorghum genome (for the bottom two rows), and the Y-axis shows the −log(10) P-values from either the unified mixed linear model (MLM; presented in the left column) or the logistic mixed model (LMM; presented in the right column). Quantile quantile (QQ)-plots depicting the observed (Y-axis) and expected (X-axis) −log(10) P-values are inserted into each Manhattan plot. (TIFF) [file pone.0207752.s010.tiff]

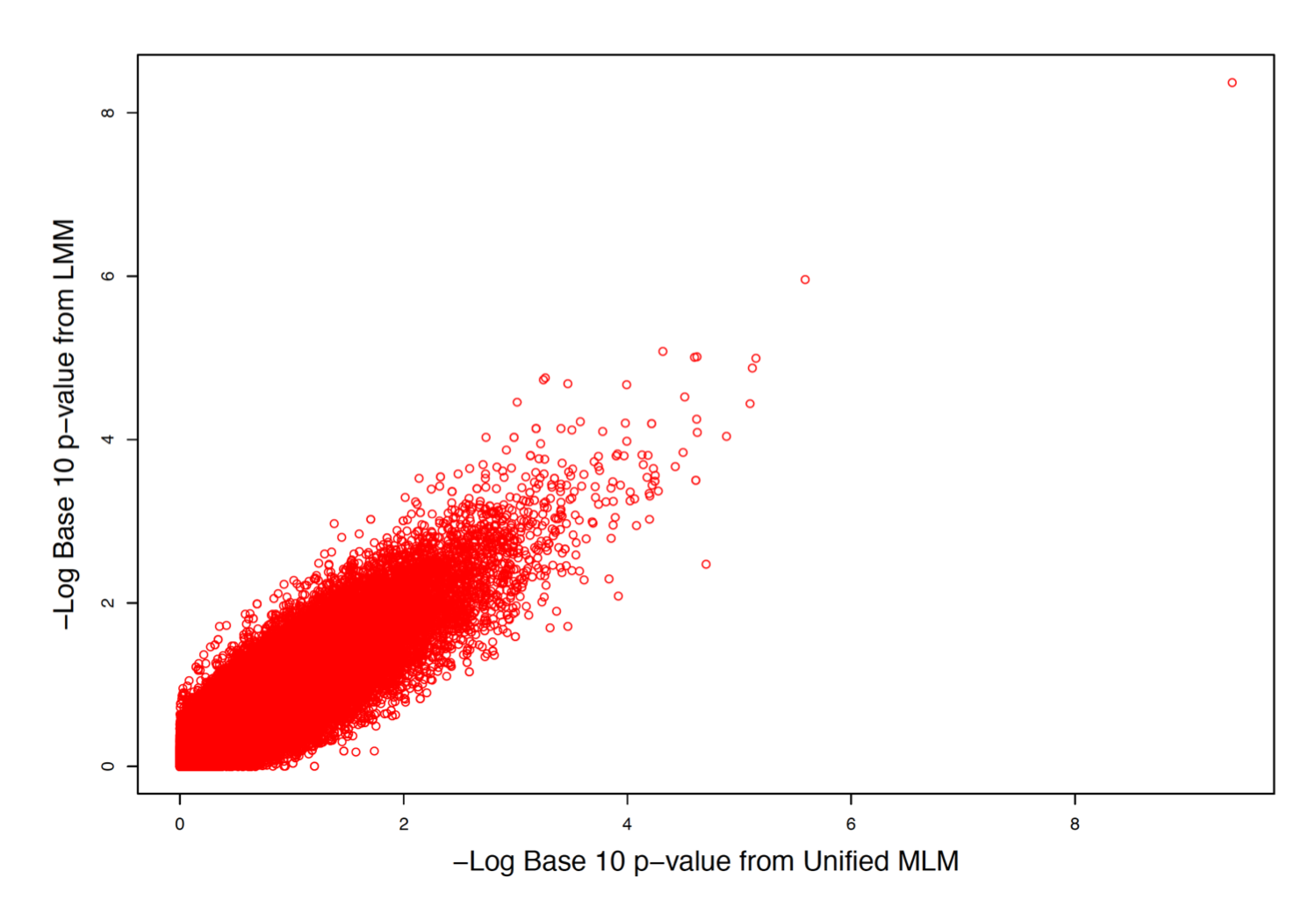

Supplement: S10 Fig — Plot of -log10(P-values) of SNPs from the logistic mixed model (Y-axis) against those from the unified mixed linear model (X-axis) for the genome-wide association study conducted for α-tocopherol levels in maize grain in the Goodman diversity panel dichotomized at the 75th percentile. Both sets of -log10(P-values) are from testing H0: no association between the tested SNP and the phenotype. (TIFF) [file pone.0207752.s011.tiff]

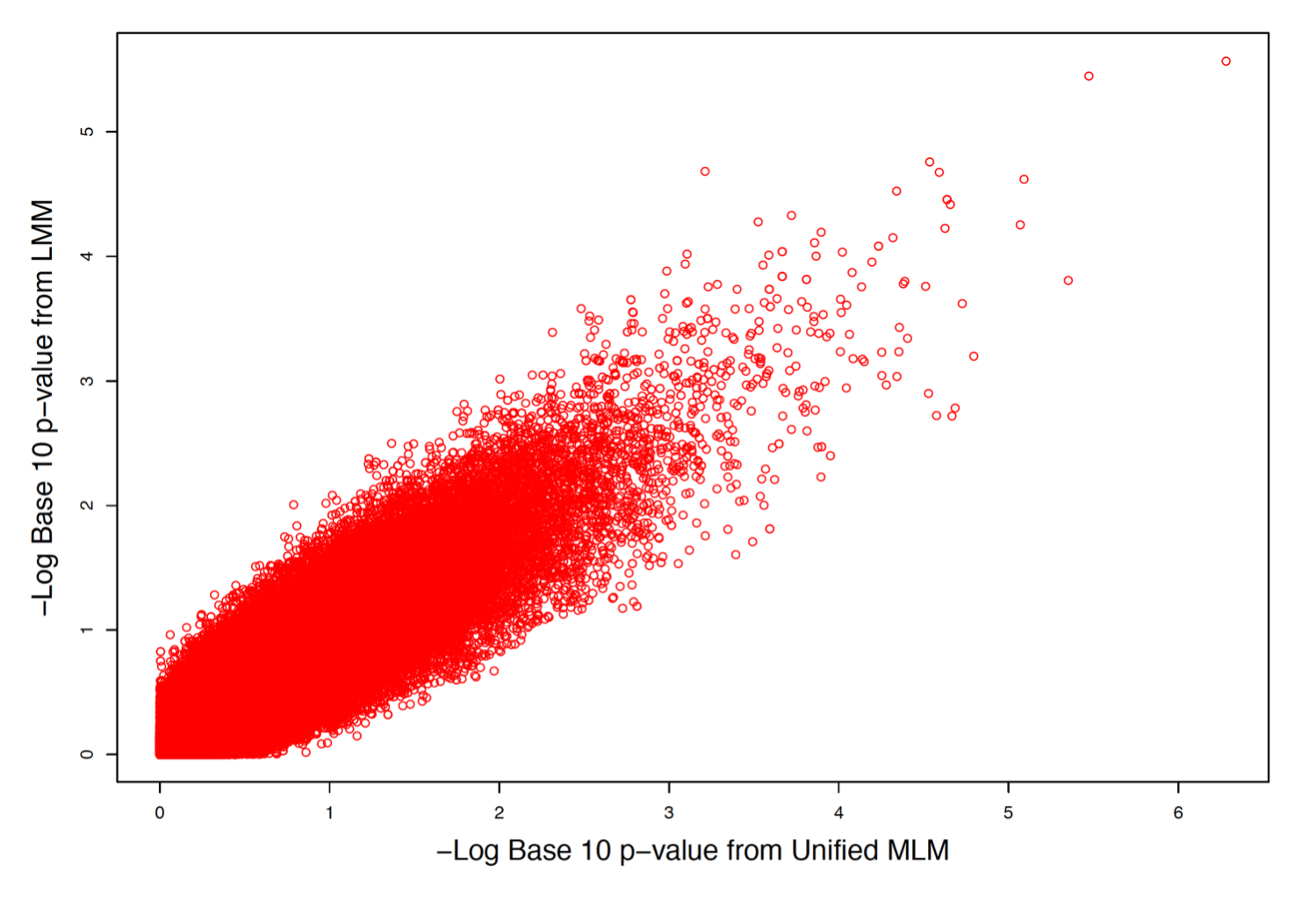

Supplement: S11 Fig — Plot of -log10(P-values) of SNPs from the logistic mixed model (Y-axis) against those from the unified mixed linear model (X-axis) for the genome-wide association study conducted for zeaxanthin levels in maize grain in the Goodman diversity panel dichotomized at the 75th percentile. Both sets of -log10(P-values) are from testing H0: no association between the tested SNP and the phenotype. (TIFF) [file pone.0207752.s012.tiff]

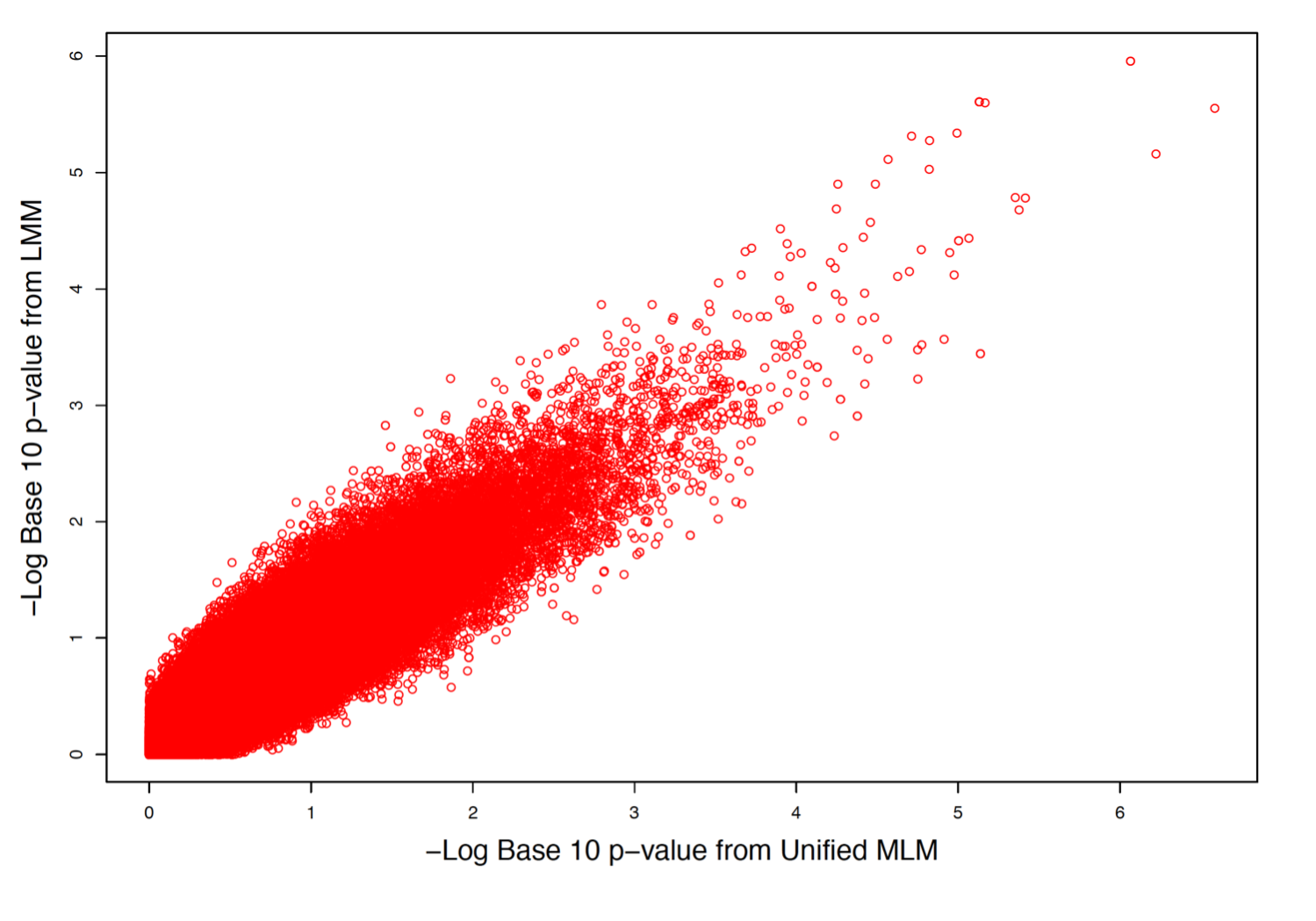

Supplement: S12 Fig — Plot of -log10(P-values) of SNPs from the logistic mixed model (Y-axis) against those from the unified mixed linear model (X-axis) for the genome-wide association study conducted for maize ear height in the Goodman diversity panel dichotomized at the 75th percentile. Both sets of -log10(P-values) are from testing H0: no association between the tested SNP and the phenotype. (TIFF) [file pone.0207752.s013.tiff]

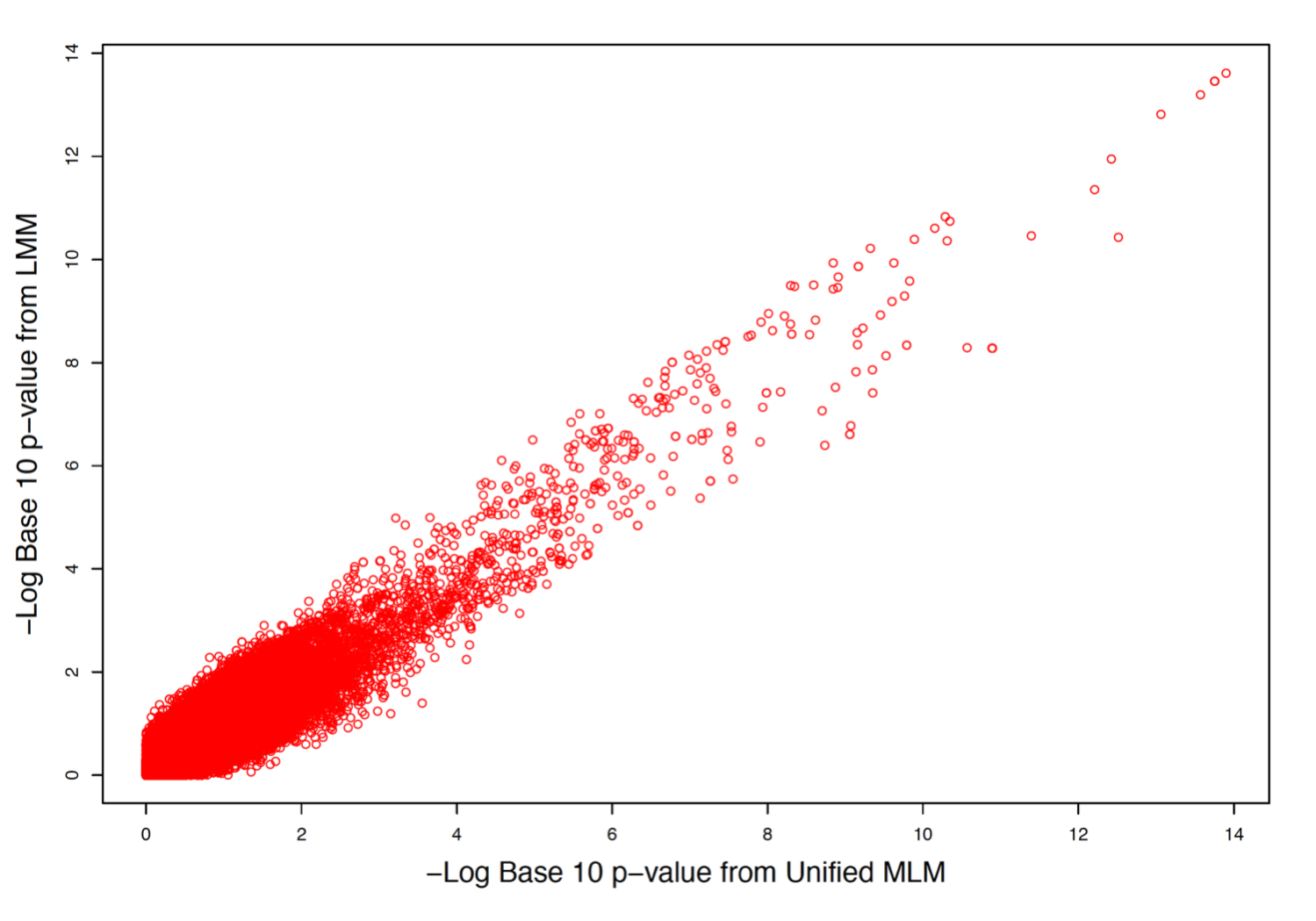

Supplement: S13 Fig — Plot of -log10(P-values) of SNPs from the logistic mixed model (Y-axis) against those from the unified mixed linear model (X-axis) for the genome-wide association study conducted for sorghum plant height in the US sorghum association panel dichotomized at the 75th percentile. Both sets of -log10(P-values) are from testing H0: no association between the tested SNP and the phenotype. (TIFF) [file pone.0207752.s014.tiff]

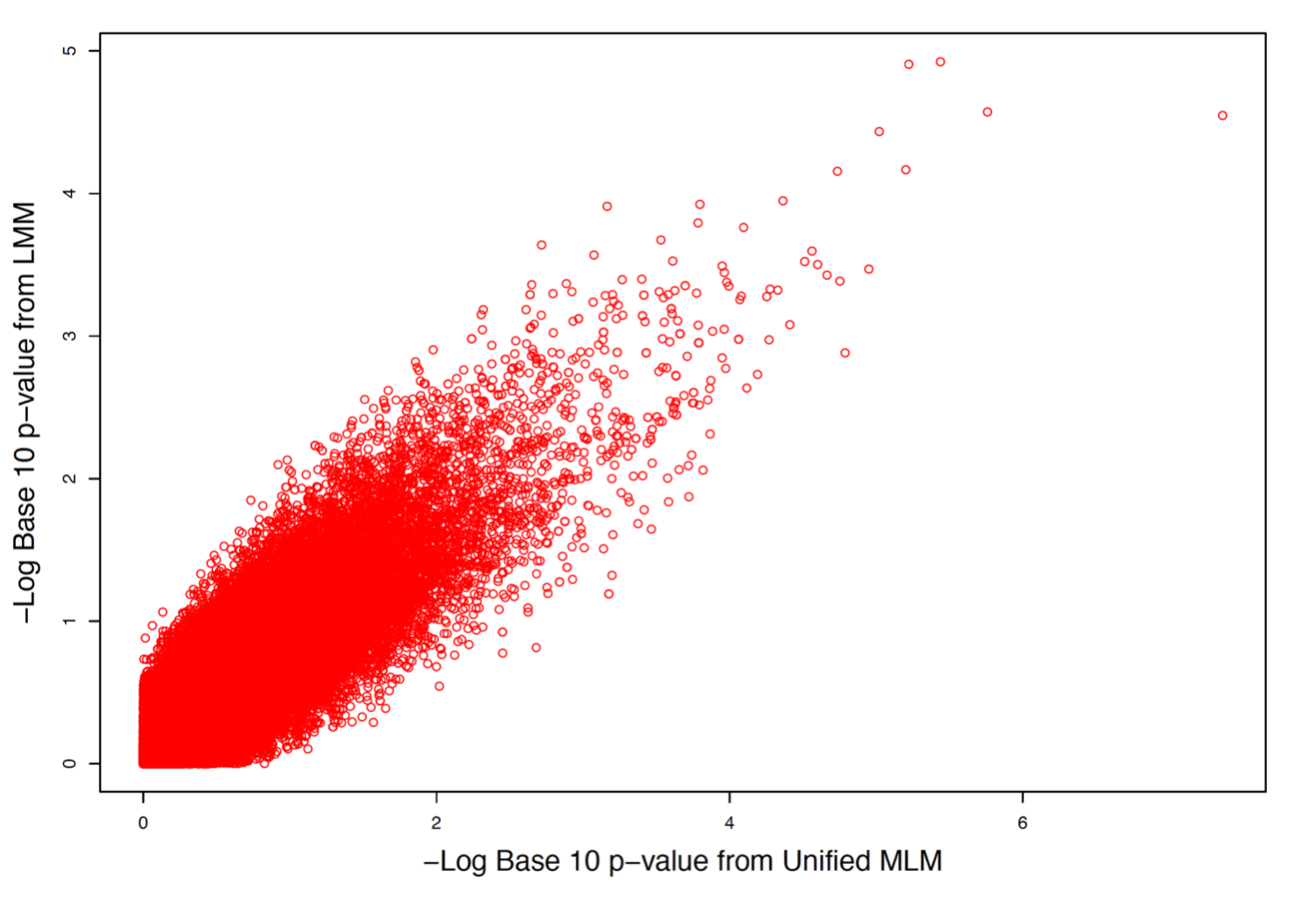

Supplement: S14 Fig — Plot of -log10(P-values) of SNPs from the logistic mixed model (Y-axis) against those from the unified mixed linear model (X-axis) for the genome-wide association study conducted for sorghum branch length in the US sorghum association panel dichotomized at the 75th percentile. Both sets of -log10(P-values) are from testing H0: no association between the tested SNP and the phenotype. (TIFF) [file pone.0207752.s015.tiff]

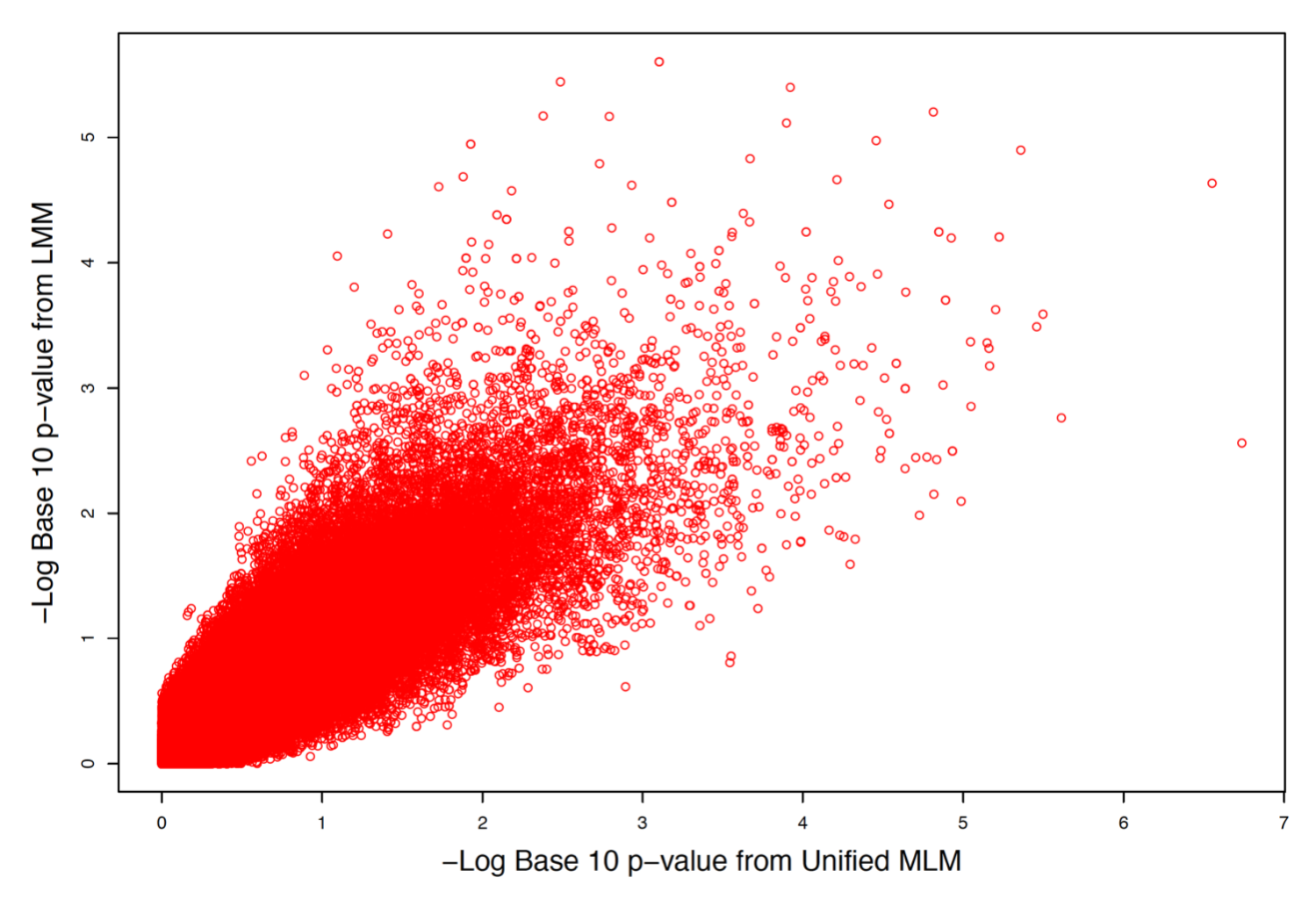

Supplement: S15 Fig — Plot of -log10(P-values) of SNPs from the logistic mixed model (Y-axis) against those from the unified mixed linear model (X-axis) for the genome-wide association study conducted for binary trait Y simulated in the Goodman maize diversity panel where P{Y = 1} = 0.5 in the non-tropical subpopulation and P{Y = 1} = 0.05 in the non-tropical subpopulation. Both sets of -log10(P-values) are from testing H0: no association between the tested SNP and the phenotype. (TIFF) [file pone.0207752.s016.tiff]

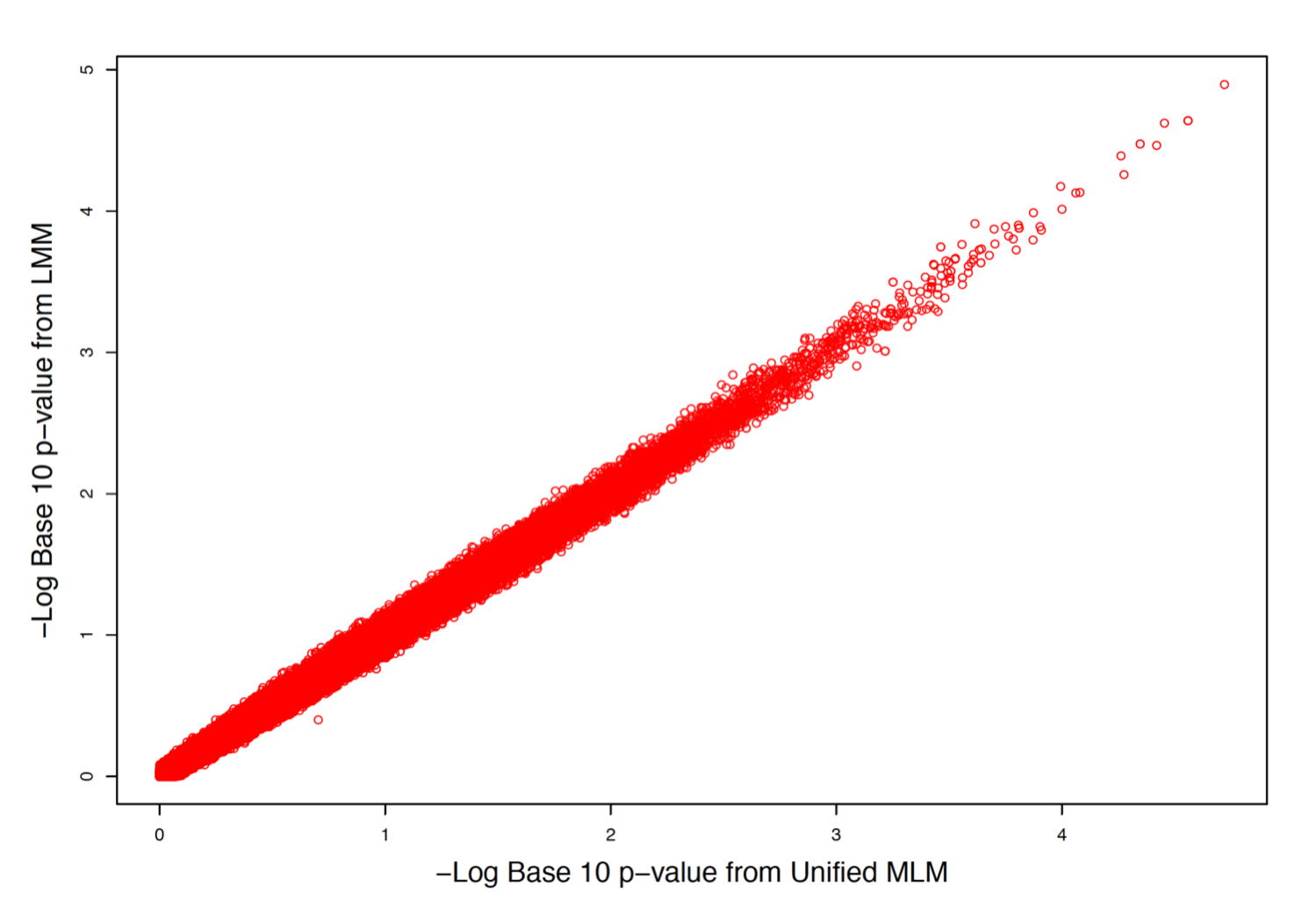

Supplement: S16 Fig — Plot of -log10(P-values) of SNPs from the logistic mixed model (Y-axis) against those from the unified mixed linear model (X-axis) for the genome-wide association study conducted for binary trait Y simulated in the Goodman maize diversity panel where the P{Y = 1} = 0.5 regardless of the subpopulation. Both sets of -log10(P-values) are from testing H0: no association between the tested SNP and the phenotype. (TIFF) [file pone.0207752.s017.tiff]
